# SURFBAT: a surrogate family based association test building on large imputation reference panels

**DOI:** 10.1093/g3journal/jkae287

**Published:** 2024-12-09

**Authors:** Anthony F Herzig, Simone Rubinacci, Gaëlle Marenne, Hervé Perdry, FrEx Consortium, Emmanuelle Génin, Dominique Campion, Jean-François Dartigues, Jean-François Deleuze, Jean-Charles Lambert, Richard Redon, Thomas Ludwig, Benjamin Grenier-Boley, Sébastien Letort, Pierre Lindenbaum, Vincent Meyer, Olivier Quenez, Christian Dina, Céline Bellenguez, Camille Charbonnier-Le Clézio, Joanna Giemza, Stéphanie Chatel, Claude Férec, Hervé Le Marec, Luc Letenneur, Gaël Nicolas, Karen Rouault, Delphine Bacq, Anne Boland, Doris Lechner, FranceGenRef Consortium, Jean-François Deleuze, Emmanuelle Génin, Richard Redon, Chantal Adjou, Stéphanie Chatel, Claude Férec, Marcel Goldberg, Philippe-Antoine Halbout, Hervé Le Marec, David L’Helgouach, Karen Rouault, Jean-Jacques Schott, Anne Vogelsperger, Marie Zins, Delphine Bacq, Hélène Blanché, Anne Boland, Robert Olaso, Pierre Lindenbaum, Thomas Ludwig, Vincent Meyer, Florian Sandron, Damien Delafoy, Lourdes Velo-Suárez, Isabel Alves, Ozvan Bocher, Christian Dina, Anthony F Herzig, Matilde Karakachoff, Gaëlle Marenne, Aude Saint Pierre, Véronique Geoffroy, Jean-François Deleuze, Christian Dina, Julien Barc, Richard Redon, Olivier Delaneau, Emmanuelle Génin

**Affiliations:** Inserm, Université de Bretagne-Occidentale, EFS, UMR 1078, GGB, Brest F-29200, France; Institute for Molecular Medicine Finland, University of Helsinki, Helsinki 00290, Finland; Inserm, Université de Bretagne-Occidentale, EFS, UMR 1078, GGB, Brest F-29200, France; CESP Inserm U1018, Université Paris-Saclay, Villejuif F-94807, France; Inserm, Université de Bretagne-Occidentale, EFS, UMR 1078, GGB, Brest F-29200, France; LABEX GENMED, Centre National de Recherche en Génomique Humaine, Evry, Paris F-91000, France; Université Paris-Saclay, CEA, Centre National de Recherche en Génomique Humaine (CNRGH), Evry F-91000, France; CEPH, Fondation Jean Dausset, Paris F-75010, France; Nantes Université, CNRS, INSERM UMR 1087, L’Institut du Thorax, Nantes F-44000, France; Nantes Université, CNRS, INSERM UMR 1087, L’Institut du Thorax, Nantes F-44000, France; Nantes Université, CNRS, INSERM UMR 1087, L’Institut du Thorax, Nantes F-44000, France; Regeneron Genetics Center, Tarrytown, NY 10591, USA; Inserm, Université de Bretagne-Occidentale, EFS, UMR 1078, GGB, Brest F-29200, France; CHU Brest, Brest F-29200, France

**Keywords:** imputation, local ancestry, shared controls, reference panel, fine-structure, population stratification

## Abstract

Genotype–phenotype association tests are typically adjusted for population stratification using principal components that are estimated genome-wide. This lacks resolution when analyzing populations with fine structure and/or individuals with fine levels of admixture. This can affect power and precision, and is a particularly relevant consideration when control individuals are recruited using geographic selection criteria. Such is the case in France where we have recently created reference panels of individuals anchored to different geographic regions. To make correct comparisons against case groups, who would likely be gathered from large urban areas, new methods are needed. We present SURFBAT (a surrogate family based association test), which performs an approximation of the transmission-disequilibrium test. Our method hinges on the application of genotype imputation algorithms to match similar haplotypes between the case and control groups. This permits us to approximate local ancestry informed posterior probabilities of un-transmitted parental alleles of each case individual. This is achieved by assuming haplotypes from the imputation panel are well-matched for ancestry with the case individuals. When the first haplotype of an individual from the imputation panel matches that of a case individual, it is assumed that the second haplotype of the same reference individual can be used as a locally ancestry matched control haplotype and to approximately impute un-transmitted parental alleles. SURFBAT provides an association test that is inherently robust to fine-scale population stratification and opens up the possibility of efficiently using large imputation reference panels as control groups for association testing. In contrast to other methods for association testing that incorporate local-ancestry inference, SURFBAT does not require a set of ancestry groups to be defined, nor for local ancestry to be explicitly estimated. We demonstrate the interest of our tool on simulated datasets, as well as on a real-data example for a group of case individuals affected by Brugada syndrome.

## Teaser

Here Herzig *et al*. present a novel statistical method for testing associations between genotypes and phenotypes in human populations. Rather than having to recruit control individuals, the authors’ method allows for publicly available panels of reference haplotypes (typically used for missing genotype imputation) to serve as shared controls.

## Introduction

Genome-wide association studies (GWAS) have become the established approach for an agnostic search of genes or genetic variants that may play a role in the development of complex multifactorial diseases (Tam *et al*. 2019). A widely accepted notion is that in a case-control design, adjustments should be made for population stratification (Price *et al*. 2006). Confounding due to population stratification is avoided when using family based association tests (Horvath *et al*. 2001; Laird and Lange 2018) where case individuals and their unaffected close relatives are both recruited to the study; hence each case individual will essentially have ancestry-matched controls. However, family based designs are currently not widely used in the study of complex trait genetics due to the difficulty of recruiting large numbers of families. Indeed, due to the cost and infrastructure required for genome sequencing, a prevalent approach is to recruit only cases and compare them to external panels of controls or to set-up large cross-sectional studies (or biobanks), which allow for the study of many different phenotypes from a single dataset. There are many examples of population-based panels that could allow for association studies to be completed without the need to recruit further control individuals; e.g. the UK biobank (Sudlow *et al*. 2015), the Estonian biobank (Leitsalu *et al*. 2015), and goNL in the Netherlands (Boomsma *et al*. 2014).

The huge sample sizes that can be achieved with case-control or biobank designs that afford statistical power for the detection of new signals is the main reason that they have become more prevalent than family based designs; but at the cost of having to hence deal with population stratification. This is usually achieved by adjusting for principal components (PCs) calculated from a genotype correlation matrix or using statistics relating to estimates of population fine-structure across the sample (Hu *et al*. 2023). Such adjustments are “global” in the sense that the PCs added to the association model use genome-wide calculations. It is possible to adjust locally, with the idea that patterns of stratification may not be equal in all genomic regions. This approach has been shown to be of interest (Qin *et al*. 2010; Zhang and Stram 2014), notably in the study of admixed populations (Wang *et al*. 2011; Duan *et al*. 2018).

Typically, such local adjustment requires local-ancestry inference to be first performed using either hidden Markov modeling (HMM) (e.g. HAPMIX (Price *et al*. 2009), LAMP (Sankararaman *et al*. 2008), or FLARE (Browning *et al*. 2023)) or recent methods that use random forests (RFMix; Maples *et al*. 2013), dynamic optimization (Loter; Dias-Alves *et al*. 2018), or neural networks (LAI-net; Montserrat *et al*. 2020). Such methods essentially color or paint (Lawson *et al*. 2012) each study individual’s haplotypes based on the similarity of haplotype segments with haplotypes in a reference set of individuals from different ancestral groups (that have to be defined at some point). The local-ancestry coloring can then be incorporated into association testing, for example through logistic regression (TRACTOR; Atkinson *et al*. 2021), mixed-modeling (asaMap; Skotte *et al*. 2019), or through joint testing of genotype and ancestry associations (Tang *et al*. 2010). Including such information has been demonstrated to enhance association studies in terms of power for discovery (Liu *et al*. 2013; Atkinson *et al*. 2021), fine-mapping (Zaitlen *et al*. 2010; Martin *et al*. 2018), and even for studies of interaction effects (Aschard *et al*. 2015). A key problem in these methods is the choice of predefined ancestry groups and estimation of local ancestry. For studies of recently admixed populations, this may be practical (Baran *et al*. 2012; Reiner *et al*. 2012; Shetty *et al*. 2012, 2015; Pino-Yanes *et al*. 2015; Spear *et al*. 2019) but in studies of populations with fine-scale population structure it will not be clear how to define different ancestry groups. For example, French populations harbor important fine-scale population structure (Pierre *et al*. 2020), which should be taken into account during association testing. Yet, it would not be clear how to best divide reference individuals into different groups, hence making local-ancestry inference problematic; a more fluid method is required.

Here, we present a new method providing the following key advantages: our method adjusts for local patterns of population stratification, but unlike existing methods that do so, there is no constraint on having to choose and define ancestry groups or explicitly map local ancestry. This is achieved by approximating a family based study design from case only data using the idea of surrogate parents (Kong *et al*. 2008) and imputation using large reference panels. This is accomplished by using HMMs (Rabiner and Juang 1986) that have been previously optimized in the domain of genotype imputation (Marchini and Howie 2010). We describe our method as a surrogate family based association test (SURFBAT).

## Methods

### Leveraging haplotype-sharing

Genotype imputation algorithms are based on the Li–Stephens model (Li and Stephens 2003) where given a large group of *n* haplotypes in a population, an $(n+1{)}^{\mathrm{th}}$ haplotype can be modeled as a mosaic of small haplotype segments or “chunks” from the pool of *n*. In isolated populations (such as Iceland), this works particularly well due to the sharing of long haplotype segments that are identical-by-descent (Thompson 2013; Herzig *et al*. 2018). The concept of surrogate parents suggests that for each given individual at a given point of the genome, even if the true parents of the individual are not present in the sample, two groups of surrogate parents can be identified who share a short haplotype that is at least very similar to the maternal or paternal haplotype of the given individual. This was originally employed for the purpose of statistical phasing (Palin *et al*. 2011), genotype imputation (Livne *et al*. 2015), and parent-of-origin analyses (Kong *et al*. 2009). Similar ideas have recently resurfaced for the same themes (Hofmeister *et al*. 2022, 2023; Noto and Ruiz 2022) now that biobank size data have become large enough. Such approaches that were previously only viable in the domain of isolated populations have thus become applicable also for nonisolated populations.

SURFBAT takes a group of case individuals and identifies surrogate parents from within a large reference panel of individuals. Crucially, we interrogate the haplotypes of the surrogates that are not shared with the case individual in question. The nonshared haplotypes between case individuals and their surrogates, we argue, represent a resource of ancestry-matched haplotypes and in the surrogate–parent interpretation represent an approximation of the un-transmitted alleles of the case individuals parents. This effectively provides a rough imputation of parental genotypes and hence an approximation of a transmission-disequilibrium test (TDT) (Spielman *et al*. 1993) can be made to test for association with a trait without sequencing parental genomes. Another interpretation of the method is that for each case individual, SURFBAT creates a pseudo-control matched on local ancestry from within the reference panel. SURFBAT performs a TDT test based on the methods that incorporate genotype uncertainty (as we are using imputed genotypes) given by Taub *et al*. 2012. SURFBAT locates and weights the contribution of surrogates using the Li–Stephens model for genotype imputation as implemented in leading software IMPUTE5 (Rubinacci *et al*. 2020).

### The SURFBAT test

In standard genotype imputation, each haplotype $\left(h_{i}\right)$ of the target group is modeled as an imperfect mosaic of the 2N haplotypes $(H_{k},k=1,\ldots ,2N)\;$ of the reference panel. This is achieved using an HMM, with states at each genomic position where a variant is observed in both the reference panel and the target group; typically, the list of positions for which the target group have been genotyped. The hidden states of the model at position j indicate which haplotype (or cluster of haplotypes) in the reference panel is providing the mosaic tile for haplotype $h_{i}$ at position *j*; often referred to as the copying state as the target haplotype will probabilistically “copy” from these reference haplotypes to achieve the missing genotype imputation. Essentially, one wishes to find the reference haplotype that shares the most recent common ancestor with the target haplotype. The observed states are the allelic values of haplotype $h_{i}$. The implementation of the HMM and the transmission and emission probabilities can slightly vary between software; globally, the transition probabilities are calculated based on the size of the reference panel and an estimated recombination rate between the genetic locations of adjacent states; and the emission probabilities allow for the mosaic to be imperfect in the sense that the target haplotype and the haplotypes in the reference panel can differ due to either recent mutations or genotyping/sequencing error.

We denote the hidden states of haplotype $h_{i}$ as $s_{i}$, which take values in 1,…,2N where *N* is the number of diploid individuals in the reference panel. The observed states (allelic values coming from genotyping data) of haplotype $h_{i}$ are denoted as $o_{i}$. The HMM provides the posterior probabilities of the hidden states at each position *j* using the forward–backward algorithm (Baum *et al*. 1970; Rabiner and Juang 1986): $P(s_{i}^{j}=k\;|\;o_{i})$. These posterior probabilities are then extended to markers, noted as $j^{\prime }$, that are not shared between the target and reference panel through linear interpolation.

The missing alleles for marker $j^{\prime }$ in haplotype $h_{i}$ are then imputed with the following dosage:


$$\rho_{i}^{\;j^{\prime }}=\;\overset{2N}{\underset{k=1}{\sum }}\;P(s_{i}^{\;j^{\prime }}=k\;|\;o_{i})1_{\left\{H_{k}^{\;j^{\prime }}=1\right\}}$$

Where the quantity $1_{\left\{H_{k}^{\;j^{\prime }}=1\right\}}$ is equal to 0 or 1 if the reference haplotype $H_{k}$ carries a major or minor allele at marker $j^{\prime }$, respectively. The final imputed dosage for individuals in the target group is simply the sum of the dosage of their two haplotypes. The dosage takes a value between 0 and 2 and represents the individual’s expected count of minor-alleles for marker $j^{\prime }$. However, here we are interested in the haplotype dosages (with values between 0 and 1). SURFBAT keeps each individual’s two haplotype dosages separate and furthermore calculates the “un-transmitted” dosages, which are simply as follows:


$$\pi_{i}^{\;j^{\prime }}=\;\overset{2N}{\underset{k=1}{\sum }}\;P(s_{i}^{\;j^{\prime }}=k\;|\;o_{i})1_{\left\{H_{r(k)}^{\;j^{\prime }}=1\right\}}$$

Assuming that the haplotypes in the reference panel are stored in groups of two for each reference panel individuals (reference haplotypes 1 and 2 correspond to the first individual, haplotypes 3 and 4 to the second individual etc.), r(k) is a simple function that gives the index of the partner haplotype to haplotype *k*. Explicitly, r(k)=k+1 if *k* is odd and r(k)=k−1 if *k* is even. We implemented the calculation of both $\rho_{i}^{\;j^{\prime }}$ and $\pi_{i}^{\;j^{\prime }}$ within the existing imputation algorithm IMPUTE5, for positions that are not genotyped (e.g. $j^{\prime }$) and that are genotyped (e.g. *j*), alike.

Note that there is an implicit yet crucial assumption that the two haplotypes within an individual on the reference panel share a relatively similar ancestry. In the context of our study, this is reasonable for the reference panels that we use (see later sections): the 1000 Genome Project panel (1000G) (The 1000 Genomes Project Consortium 2015) and the FranceGenRef (FGR) panel (Alves *et al*. 2024; Herzig *et al*. 2024). The individuals of FGR were recruited based on grandparent birthplace data taking individuals with all four grandparents born within a small locality; hence, approximately ensuring that such individuals have both maternal and paternal haplotypes from a similar ancestry. Indeed, such a recruitment strategy has often been used for the construction of other reference panels (Leslie *et al*. 2015; Bycroft *et al*. 2019; Raveane *et al*. 2019). Therefore, when the reference panel has such a construction, haplotype $H_{r(k)}$ is approximately matched (in terms of ancestry) to haplotype $H_{k}$. Imputation panels containing recently admixed individuals, where $H_{r(k)}$ would not be well-matched to $H_{k}$, would therefore add unwanted stratification to our test and hence should be avoided if possible.

When using IMPUTE5 with the specifically designed option “–surfbat,” at marker $j^{\prime }$ for target individual *i* (with haplotypes $i_{1}$ and $i_{2}$), the four dosages $\rho_{i_{1}}^{\;j^{\prime }}$, $\rho_{i_{2}}^{\;j^{\prime }}$, $\pi_{i_{1}}^{\;j^{\prime }}$, and $\pi_{i_{2}}^{\;j^{\prime }}$ are calculated and reported. In the “surrogate family” interpretation of the four dosages, individual *i* has received two alleles with expected values $\rho_{i_{1}}^{\;j^{\prime }}$, and $\rho_{i_{2}}^{\;j^{\prime }}$ from their parents and the corresponding un-transmitted alleles from the parents have expected values $\pi_{i_{1}}^{\;j^{\prime }}$ and $\pi_{i_{2}}^{\;j^{\prime }}$. We refer to these last two expected values as the “pseudo-control” dosages. Then using similar notation to Taub *et al*. (2012), we form the following test-statistic for equilibrium of transmission at marker $j^{\prime }$:

First, the quantities NUM and DEN are calculated:


$$\mathrm{NUM}=\underset{i}{\sum }\;\rho_{i_{1}}^{{\;j}^{\prime }}(1-\pi_{i_{1}}^{{\;j}^{\prime }})+\rho_{i_{2}}^{{\;j}^{\prime }}(1-\pi_{i_{2}}^{{\;j}^{\prime }})$$

$$\mathrm{DEN}\;=\;\mathrm{NUM}+\underset{i}{\sum }\;\pi_{i_{1}}^{{\;j}^{\prime }}(1-\rho_{i_{1}}^{{\;j}^{\prime }})+\pi_{i_{2}}^{{\;j}^{\prime }}(1-\rho_{i_{2}}^{{\;j}^{\prime }})$$

Then, we calculate: $X=\;\frac{{\hat{\beta }}^{2}}{\mathrm{var}(\hat{\beta })}$, where $\hat{\beta }=\mathrm{logit}(\frac{\mathrm{NUM}}{\mathrm{DEN}})$, and $\mathrm{var}(\hat{\beta })=\frac{\mathrm{DEN}}{\mathrm{NUM}(\mathrm{DEN}-\mathrm{NUM})}$. *X* asymptotically follows a χ^2^ distribution with 1 degree of freedom under the null hypothesis of equilibrium of transmission. This corresponds to an exact-form test-statistic for conditional likelihood regression (Schwender *et al*. 2012).

We also provide an alternative test where the cases and pseudo-controls are not paired; a simple test of marginal homogeneity (Bickeböller and Clerget-Darpoux 1995) from a contingency table of the expected allelic values of the haplotypes of the case individuals and of the pseudo-controls. The test is constructed from a simple 2-by-2 contingency table with cells A, B, C, and D with marginal totals $n_{1.}=A+C$, $n_{2.}=B+D$, $n_{.1}=A+B$, $n_{.2}=C+D;$ the four cells A–D are calculated as follows: $A=\underset{i}{\sum }\;(1-\rho_{i_{1}}^{{\;j}^{\prime }})(1-\pi_{i_{1}}^{{\;j}^{\prime }})$ + $(1-\rho_{i_{2}}^{{\;j}^{\prime }})(1-\pi_{i_{2}}^{{\;j}^{\prime }}),\;B=\underset{i}{\sum }\;(1-\rho_{i_{1}}^{{\;j}^{\prime }})\pi_{i_{1}}^{{\;j}^{\prime }}$+ $(1-\rho_{i_{2}}^{{\;j}^{\prime }})\pi_{i_{2}}^{{\;j}^{\prime }},\;\;$$C=\underset{i}{\sum }\;\rho_{i_{1}}^{{\;j}^{\prime }}(1-\pi_{i_{1}}^{{\;j}^{\prime }})$ + $\rho_{i_{2}}^{{\;j}^{\prime }}(1-\pi_{i_{2}}^{{\;j}^{\prime }})$, and $D=\underset{i}{\sum }\;\rho_{i_{1}}^{{\;j}^{\prime }}\pi_{i_{1}}^{{\;j}^{\prime }}$ + $\rho_{i_{2}}^{{\;j}^{\prime }}\pi_{i_{2}}^{{\;j}^{\prime }}$. The statistics A to D that comprise the contingency table can be readily interpreted as the expected sums of different configurations and transmission statuses of parental alleles.

Then, $X=\;\underset{m=1,2}{\sum }\;\frac{{\left(n_{m.}-n_{.m}\right)}^{2}}{\left(n_{m.}+n_{.m}\right)}$ again asymptotically follow a χ^2^ distribution with 1 degree of freedom under the null hypothesis of marginal homogeneity.

In order to perform SURFBAT, only genotyping array data for the case individuals are required, along with WGS data for the control individuals who must be formed into a phased imputation reference panel. The cases are imputed against the reference panel using IMPUTE5 and the “–surfbat” flag activated, which also calculates the per-SNP test-statistics and corresponding *P*-values. There is the possibility to place thresholds on the minor-allele frequency (MAF) and the imputation quality (INFO score), with default settings placed at 0.01 and 0.3, respectively. This is due to the fact that for rare variants and poorly imputed variants the test is not appropriate; as is the case for a traditional GWAS using imputed data.

### Creating a reference panel

In this study, we took the haplotype reference panel for the 1000G (Phase 3), as was made available by imputation software IMPUTE2 (Howie *et al*. 2011). This dataset was merged with the whole genome sequencing (WGS) data of the FGR project. For certain analyses, we retained only the 404 non-Finnish Europeans (NFE) from the 1000G panel. In order to prepare the FGR data, we used the sequencing data quality control pipeline RAVAQ (Marenne *et al*. 2022), and phased the data using SHAPEIT4 (Delaneau *et al*. 2019). Merging and manipulation of the data files was achieved using the R-package “gaston” (Perdry *et al*. 2018), plink v1.9 (Purcell *et al*. 2007), bcftools (Danecek *et al*. 2021), SHAPEIT5 (Hofmeister *et al*. 2023), and pbwt (Durbin 2014). Variants with an extreme difference in MAF between FGR and the NFE 1000G individuals were removed from the control panel as these were deemed to likely represent a batch effect between the sequencing data of FGR and the public 1000G data. Specifically, we calculated the Hudson’s Fst (Hudson *et al*. 1992; Bhatia *et al*. 2013) statistic between FGR and NFE and excluded variants with a statistic greater than the observed mean plus 6 times the observed standard deviation. The combined reference panel of 1000G and FGR will be labeled as 1000G + FGR, and the panel than combines NFE and FGR as NFE + FGR. These panels will be used in this work for the simulation studies and a real-data example. 1000G + FGR represents 6,724 haplotypes attained by merging the 2,504 individuals from the 1000G project (5,008 haplotypes) with 856 individuals with WGS data from FGR (1,716 haplotypes). The final panel involves 10,252,495 bi-allelic variants across the 22 autosomal chromosomes and 6,724 haplotypes. For certain analyses, we restricted the 1000G to the NFE samples, to form the reference panel NFE + FGR across the same set of variants and containing 2,520 haplotypes.

### Simulation studies

Our first simulated study involved simulating a group of 450 case individuals who were constructed as mosaics of the control group (1000G + FGR) using the R-package “Mozza” (https://github.com/genostats/Mozza), which leveraged the Li–Stephens Markov process of forming new mosaic haplotypes. To keep calculation time short, we only simulated chromosomes 10–22 and each chromosome was simulated separately. At each instance when a mosaic tile was drawn for a new case individual, we assigned a 50% chance to copy from a haplotype from the African continent super population from 1000G (AFR) and 50% from FGR. A tile length of 10 centi-Morgans (cM) was used for the simulation. We simulated a pool of 40,000 haplotypes; our 450 simulated case individuals’ 900 haplotypes were sampled from this pool in order to create two association signals on chromosomes 11 and 20. On chromosome 11, we sampled an excess of haplotypes with tiles copied from FGR haplotypes overlapping base-pair position 66,602,100 (near the middle of the chromosome); simply be choosing 650 simulated chromosomes carrying a tile copied from FGR and 250 with a tile copied from AFR to give a total of 900 haplotypes required to simulate 450 cases. On chromosome 20, we sampled an excess of FGR haplotypes that carry an alternative allele for the common variant rs197819; among the 900 simulated haplotypes for the case individuals there were 500 carriers of such haplotypes containing the alternative allele and 400 noncarriers, which represents far more carriers than when haplotype segments are drawn at random with Mozza. This variant (rs197819) was chosen at random among a set of candidate variants on chromosome 20 with at least a MAF of 0.2 in both AFR and FGR. Genotyping data for the case group was simulated by extracting positions from the UK biobank SNP-array (details here: https://biobank.ndph.ox.ac.uk/showcase/refer.cgi?id=149601). We simulated 450 cases in order to have enough power to demonstrate adequately the performance of SURFBAT while also presenting a scenario not too dissimilar to the real-data example involving 346 cases (see following section).

For the more elaborate simulation set-up using msprime (Baumdicker *et al*. 2022) and the standard library of population genetic models (Adrion *et al*. 2020), we used the following command: *stdpopsim HomSap-c chr15-o afr-america-chr15.trees-s 1213-g HapMapII_GRCh38-d AmericanAdmixture_4B11 AFR:15000 EUR:15000 ASIA:15000 ADMIX:2000*. This simulated a sequence of coalescent trees describing 47,000 individuals from the American Admixture scenario (Browning *et al*. 2018): 15,000 each from African, European, and Asian Ancestry groups, plus 2,000 3-way admixed individuals. The simulated admixture event occurred 12 generations ago with proportions of 1/6, 1/3, and 1/2 for African, European, and Asian components, respectively. The sequence of coalescent trees simulated in this way was loaded into python with the package “tskit” (Kelleher *et al*. 2018), which was also used to create a simulated variant-call file (VCF) of phased haplotypes. As this simulated data would be used for repeated simulations to explore the SURFBAT test under the null hypothesis of no association, we restricted to only chromosome 15 to avoid lengthy computations. The simulated data included 3,641,053 variants, which are more than necessary for the purposes of the simulations so we simply retained every fifth variant and also excluded any sites with a derived-allele frequency below 0.05 or above 0.95. The final simulated VCF file thus only contained a far more manageable 33,388 variants, suitable for the purposes of the simulation. To simulate array data for the purposes of SURFBAT, we restricted to variants with a minor-allele frequency above 0.1, performed linkage disequilibrium thinning with the R-package “gaston” with a threshold of $R^{2}$ = 0.8, and then randomly selected 5,000 variants. For a given simulation, the case individuals would have only phased data at these 5,000 sites and would be imputed against phased reference panels containing all 33,388 sites in order to perform the SURFBAT test.

In the simulation scenarios where the admixed individuals were used as cases and the nonadmixed individuals as controls (see Supplementary Fig. 1), SURFBAT was compared with standard GWAS (logistic regression) and to TRACTOR (Atkinson *et al*. 2021). For the GWAS, the case and control individuals would have all 33,388 variants and the association tests would be adjusted on 6 PCs. For the TRACTOR method, given that we essentially know the local ancestry of the control individuals (from populations AFR, EUR, and ASIA), we ran local-ancestry estimation using FLARE (Browning *et al*. 2023) for the 2,000 admixed individuals using the 45,000 controls as a reference group. FLARE was run with default parameters aside from setting the number of generations to 12 (as this is what was simulated by stdpopsim). We then ran logistic regression models including local-ancestry, global ancestry, and local-ancestry specific allele counts as presented in (Atkinson *et al*. 2021) in order to compute the likelihood-ratio test-statistics of TRACTOR. We also again used Mozza to simulate case individuals as mosaic haplotypes of the simulated European ancestry group, with a tile length of 5 cM.

### Brugada study

We took 346 individuals, with whole-sequencing data, affected with Brugada syndrome as a real-data example. For more information on the Brugada phenotype, we direct the reader to Barc *et al*. 2022 (referred to hereon as “Barc22”). These individuals were recruited in France and so we can assume that they can be accurately imputed using a reference panel built of FGR and 1000G (Herzig *et al*. 2024). To perform GWAS, we analyzed all common bi-allelic variants (MAF above 0.01) that were present in our total pool of reference haplotypes (1000G + FGR). GWAS was carried out in the R-package “gaston,” using logistic regression adjusted for 6 PCs; PCs were calculated from pruned data and this was also achieved using “gaston.” To apply SURFBAT to the Brugada case individuals, we required only genotyping data for these individuals. We simulated this scenario by extracting positions from the UK BioBank SNP-array from the WGS data for the 346 individuals; this array was designed to facilitate genotype imputation and hence would be a logical choice for future applications of SURFBAT. The data from the extracted array positions was phased with SHAPEIT4 and then supplied to IMPUTE5 with the required option (−surfbat) in order to calculate genome-wide SURFBAT *P*-values. Finally, to demonstrate the interest of the concept of using an imputation reference panel as a control panel, we simulated a scenario where we have both case and control individuals with genotyping array data. In this circumstance, both cases and controls were imputed and a GWAS was performed on the imputed dosages. For this, we took control individuals from 569 individuals of the FrEx database (Dartigues *et al*. 1991; 3C Study Group 2003) (http://lysine.univ-brest.fr/FrExAC/) who have genotyping data (Illumina OmniExpressExome array). Positions corresponding to this array were extracted from the 346 case individuals and we then phased (SHAPEIT4) and imputed (IMPUTE5) the 346 case and 569 control individuals together. GWAS was then performed on the imputed dosages using SNPTEST (Marchini *et al*. 2007; Wellcome Trust Case Control Consortium 2007); again using logistic regression adjusted for 6 PCs. Both 1000G + FGR and NFE + FGR were used as reference panels in the analyses of the Brugada dataset.

## Results

### Simulation studies

**Fig. 1.**
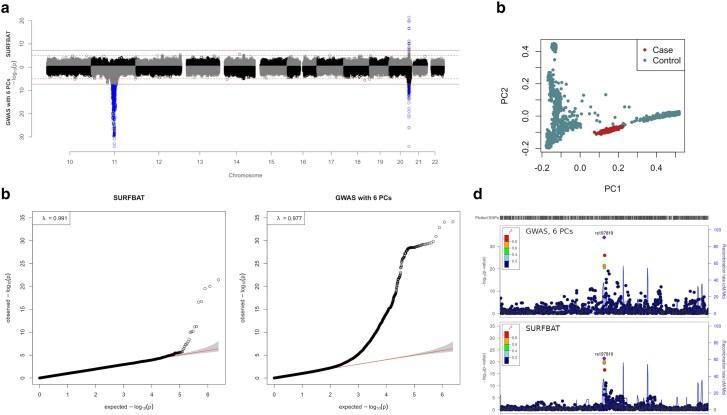
a) Miami plot of association studies either carried out using a standard GWAS (logistic regression) adjusted for 6 PCs (bottom), or with SURFBAT (top). Full horizontal lines indicate genome-wide significance (5e−8), dashed horizontal lines indicate the suggestive threshold (1e−5). b) Quantile-quantile plots (qq-plots) pertaining to the *P*-values displayed in a). c) PCs 1 and 2 estimated on the simulated cases and controls (individuals of the 1000 Genomes project and FGR). d) LocusZoom of the signal on chromosome 20 for the two methods.

We compared SURFBAT against a traditional GWAS adjusted on 6 PCs (Fig. 1) using a simple simulation set-up to demonstrate the properties of our method. To this end, we constructed a group of 450 case individuals as mosaics of the reference panel 1000G + FGR on a short genome (chromosomes 10–22). The case individuals have an admixed ancestry profile with 50% of their chromosome mosaic tiles coming from the AFR populations of 1000G (African continent) and the other 50% from the French samples of FGR. We added a signal of purely local ancestry on chromosome 11 by simulating an excess of FGR haplotypes overlapping a randomly chosen base-pair position (66,602,100) near the middle of the chromosome, but without any specific risk alleles being chosen, but hence leading to an association between local ancestry and disease status in the simulation. We also simulated a genetic risk factor signal on chromosome 20, with an excess of FGR haplotypes carrying the alternative allele for the variant rs197819 (chosen randomly). Here on chromosome 20, haplotypes containing the disease-causing allele (alternative allele for variant rs197819) are sampled both from FGR and AFR but with an excess of FGR haplotypes (see Methods for full details) and hence creating associations between the following three things 3: the observed alleles for rs197819, local ancestry, and disease status.

Using these simulated data, we observe the two key properties of SURFBAT. First, local ancestry is adjusted for and hence the strong GWAS signal on Chromosome 11 disappears. Second, the association signal on chromosome 20 is detected by SURFBAT and is also noticeably more precise as we deliberately combined the association with rs197819 with an association with local ancestry; arising from the aforementioned simulation of an excess of haplotypes segments from FGR that carry minor-alleles at rs197819 for the case individuals (see Methods for full details). This is observed in the LocusZoom (Pruim *et al*. 2010) plots in Fig. 1d. Indeed, with a direct GWAS, there are SNPs that surpass genome-wide significance up to 0.5 Mb away from rs197819; whereas SURFBAT only gives significant *P*-values to variants close to rs197819.

We explored the behavior of the SURFBAT test with a more elaborate simulation set-up using msprime (see Methods). This simulation differs crucially to the simple simulation used for Fig. 1 in that the case individuals are no longer perfect mosaics of the reference individuals and hence presents a more realistic and greater challenge for SURFBAT. SURFBAT was applied on the 2,000 admixed individuals (to be interpreted as the case individuals) with a reference panel of the remaining 45,000 nonadmixed individuals with African, European, or Asian ancestry. Smaller reference panels (subsets of 45,000) of different sizes were also used (Supplementary Fig. 1). Here, the null model for our test states that there is no different in allele-frequency between the imputed dosages of the case individuals and the imputed “pseudo-control” dosages that approximate the un-transmitted alleles of the case individuals. This null model should be respected as the case group should be well represented by mosaics of the reference panel. When the reference panel is relatively small (900 individuals split evenly between African, European, and Asian ancestry groups) there is however a clear inflation of the SURFBAT test-statistics. This however is alleviated by increasing the reference panel size (going up to all 45,000 simulated individuals with African, European, and Asian ancestry). We also observe that a traditional GWAS approach adjusted for PCs will fail to avoid inflation as local ancestry is not modeled for. As expected, TRACTOR performs slightly better than GWAS adjusted on PCs in this simulation but has the key inconvenience of having to specify the ancestry groups; this is simple in this simulation but may be less so in real-data scenarios. This serves to show that SURFBAT achieves a modeling of local ancestry with the advantage of never having to assign ancestry labels to a reference panel.

To further investigate how the reference panel composition impacts the distribution of test-statistics, we performed a simulation to approximate the scenario of the real-data example (see following section on Brugada syndrome). Here, 500 case individuals are simulated as mosaics (again using Mozza) of the 15,000 simulated individuals with European ancestry from the pool of 15,000 created using msprime. The SURFBAT test is then performed against reference panels that contain either 1,000, 2,000, or 5,000 European ancestry individuals and varying numbers of African and Asian ancestry individuals. For each realization, we retained the inflation factor (*λ*); results are given in Supplementary Fig. 2. When there are relatively few haplotypes well-matched to the case group (in this case European ancestry) in the reference panel, the test will became inflated if the reference panel also contains many not well-matched haplotypes which will nevertheless often be used for imputation (Dekeyser *et al*. 2023). This is however controlled for by including greater numbers of well-matched haplotypes. This gives the HMM of the imputation software more opportunities to find haplotype matches from the most relevant parts of the ancestry spectrum. The results of Supplementary Figs. 1 and 2 demonstrate for which scenarios the application of the SURFBAT test would be appropriate. As the test relies on imputation algorithms, what is seen is that when the imputation panel does not provide a high performance of imputation (i.e. when it the panel is too small or contains relatively few well-matched haplotypes) the test-statistics of SURFBAT will likely be inflated.

### Real-data example: Brugada syndrome

We examined an example of real data using 346 cases with Brugada syndrome with WGS data. These individuals were recruited in France and comprise part of the case group involved in the largest GWAS to date for Brugada syndrome (Barc22). Here, we used as a reference panel NFE + FGR: a combination of the NFE of 1000G (404 individuals) and FGR. NFE comprises individuals from the 1000G populations Iberian populations in Spain, Toscani in Italy, Utah residents with Northern/Western European ancestry, and British from England and Scotland (see The 1000 Genomes Project Consortium 2015 for details). The logic behind the choice of restricting the 1000G to NFE for this section is as follows: first, from experience in previous work, this group has proved to be a good reference population when analyzing and performing quality control of data from French populations (Marenne *et al*. 2022). Individuals from the fifth European population of 1000G, FIN, from Finland have slightly divergent allele frequencies and indeed the reference database gnomAD (Chen *et al*. 2024) specifically provides Finnish and non-Finnish allele frequencies for European populations. Restricting to just the NFE group for this work relates to the possibility for inflation in the SURFBAT test when the reference panel is both not sufficiently large and contains many individuals from populations that are relatively distant to the case individuals; as could be seen in certain simulation scenarios. We could confirm this by applying SURFBAT on the Brugada data but using the entire 1000G + FGR panel rather than NFE + FGR, inflation was indeed observed (Supplementary Fig. 3). Using the whole of the 1000G represents a similar scenario to the simulation results in Supplementary Fig. 2, in particular the scenarios of 500 case individuals and a reference panel containing only 1,000 population-specific individuals along with many nonspecific individuals. An intuition as to why the inflation arose is that haplotypes that are globally not well-matched to the case individuals were too often being used for imputation, and hence the haplotypes selected to form the “pseudo-controls” are drawn from pools of haplotypes that are also not always well-matched to the case group, leading to population stratification inflating the test-statistics.

**Fig. 2.**
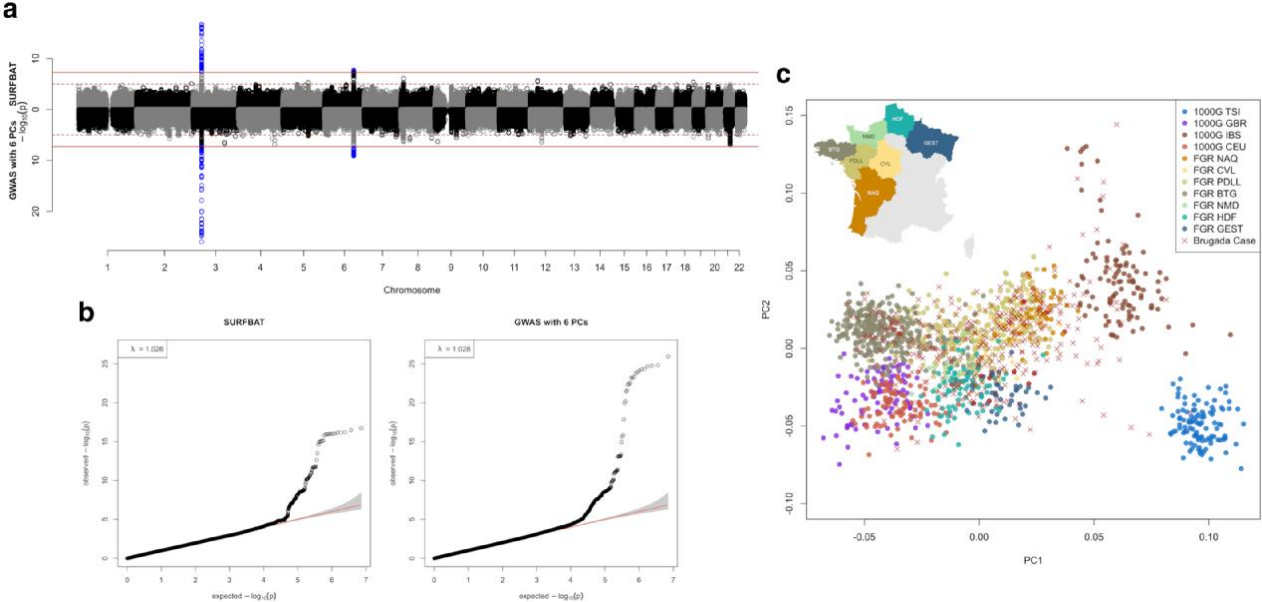
Association analyses of the Brugada dataset. a) Miami plot comparing a GWAS: 346 Brugada-case individuals with 1,260 control individuals (NFE + FGR) adjusted for 6 PCs and where both cases and controls have WGS data (bottom); against SURFBAT: 346 Brugada-case individuals with only genotyping array data are compared with the WGS data control group (NFE + FGR) (top). Results are given for a set of 7,209,684 variants; having excluded variants with a minor-allele frequency below 1% in the case group or with an imputation “info” score below 0.3. Full horizontal lines indicate genome-wide significance (5e−8), dashed horizontal lines indicate the suggestive threshold (1e−5). b) qq-plots pertaining to a). c) PCA of all individuals involved, round points denote the control individuals with colors pertaining to different populations within Europe and between different regions in France for the FGR samples: TSI, Toscani in Italy; CEU, Utah residents with Northern/Western European ancestry; GBR, British from England and Scotland; IBS, Iberian populations in Spain; NAQ, Nouvelle Aquitaine; BTG, Bretagne; NMD, Normandy; HDF, Hauts de France; PDLL, Pays de la Loire; GEST, Grande Est. An inset of the map of France is added to show the locations of the different French regions. Brugada case individuals are marked with crosses.

Having chosen a reference panel (NFE + FGR) for the Brugada analyses, three different possible scenarios of an association study were tested. (1) We performed a GWAS, adjusted on PCs (6 to match the GWAS in Barc22), against the aforementioned reference panel of NFE + FGR. (2) We applied SURFBAT, using NFE + FGR as the reference panel and “array-like” genotype data extracted from the WGS of 346 Brugada-case individuals. (3) A GWAS of the 346 case individuals against 569 French controls from the FrEX cohort. In the third scenario, array(-like) genotype data were imputed using NFE + FGR as a reference panel for both the Brugada-case group and FrEx control-group. In Fig. 2, the results of (1) and (2) are compared, and the results of (1) and (3) are compared in Supplementary Fig. 4. As mentioned above, we also applied strategy (2) using the whole 1000G + FGR panel and the results are included in Supplementary Fig. 3. SURFBAT is able to capture similar results to the direct GWAS against NFE + FGR. The third scenario involving FrEx is arguably weaker than SURFBAT, noticeably because the association peak on chromosome 6 (HEY2, NCOA7) does not attain genome-wide significance. In general, all three methods gave broadly similar results, which demonstrates the ability of SURFBAT to achieve good association testing results with only array data for the cases and access to external controls in the form of an imputation reference panel. The reference panel of NFE + FGR was well-matched to the case group in this real-data example, as shown by the PCs analysis given in Fig. 2c; note that the *y*-axis in Fig. 2c is truncated to improve the visualization, 3 case individuals in fact exceed the limits of the plot, and the full plot is given in Supplementary Fig. 5.

Concentrating on the largest association peak on chromosome 3, SURFBAT appeared to have less power compared with the GWAS strategy and the FrEx GWAS strategy. For the direct GWAS against FGR + NFE, this is not too surprising as the sample size is much higher (1,260 controls compared with SURFBAT, which uses 346 pseudo-controls). However, this direct GWAS strategy is not necessarily a realistically attainable study design and a fairer comparison for GWAS might be the FrEx GWAS strategy. The lead variant for the peak on chromosome 3 in Barc22 is rs6801957 and in Supplementary Fig. 6, we examine the power of SURFBAT against different scenarios based on the FrEx GWAS strategy by down-sampling the number of individuals. This demonstrates the intuition that SURFBAT applied to K cases will have a similar power to detect association as a GWAS involving K cases and K controls. The analysis also demonstrates that in the case of the Brugada study, genotyping all 346 cases and 0 controls and using SURFBAT gives superior power compared with approaches that might, for example, split a research budget between genotyping both cases and controls (173 individuals in each group).

In Supplementary Table 1, the *P*-values for leading SNPs from the GWAS of Barc22 (see table 1 in Barc22) are given and compared against our results. The *P*-values from the association studies in Supplementary Fig. 3 are also included in Supplementary Table 1. As we have a far smaller sample size (Barc22 analyzed 2,820 cases and 10,001 controls compared with our 346 cases and 1,260 controls), we did not have the power to detect genome-wide significant signals aside from the two most significant at SCN10A on chromosome 3 and HEY2,NCOA7 on chromosome 6. We can however discern in Fig. 2 the suggestive peak on chromosome 8, which corresponds to GATA4. As shown in Supplementary Table 1, the association analyses that we performed, including SURFBAT, were however able to replicate (*P* < 0.05) many other GWAS hits from Barc22. It is also worth noting in Fig. 2 that the GWAS strategy gives a near significant signal on chr21, more significant than GATA4, but in a noncoding region and with no apparent coherence with Barc22 and so potentially a false positive.

## Discussion

Here, we have presented a novel method, SURFBAT, for association testing for common genetic variants with binary phenotypes. SURFBAT is a case-only association test that leverages imputation panels to estimate possible case parental un-transmitted allele dosages; hence with a test-statistic derived from the literature of family based studies. This allows SURFBAT to adjust for population stratification by design, and not just at a global level but down to a local ancestry level and hence should be an interesting tool for the study of admixed individuals. This is shown in the simulation study where a large GWAS peak (chromosome 11) coming only from local ancestry completely disappears using SURFBAT while a “true” peak (chromosome 20) is maintained. Importantly, local ancestry is taken into account without having to specify a number of ancestry groups or to map local ancestry. Furthermore, the signal on chromosome 20 was much cleaner than in the GWAS suggesting that SURFBAT could aid in studies of fine-mapping.

The composition of an imputation reference panel is a very important consideration for genotype imputation (Dekeyser *et al*. 2023) and is therefore also important for SURFBAT. Supplementary Figs. 1 and 2 broadly show that in scenarios where the reference panel does not contain sufficient haplotypes in order to achieve a high quality of imputation, the SURFBAT test can falter and become inflated. In Supplementary Fig. 1, when the reference panel was too small, the admixed case individuals were not imputed with the correct 1/6, 1/3, 1/2 proportions of African, European, and Asian components and hence neither were the pseudo-controls created by SURFBAT. Similarly, in Supplementary Fig. 2, when too few well-matched haplotypes were available, more distant haplotypes were used for imputation due to unexpected matching (Howie *et al*. 2011) and this was also reflected in the pseudo-controls. Note that these issues are not necessarily an important problem regarding missing-genotype imputation of the case individuals but essentially can create population stratification within the SURFBAT test as the pseudo-controls are constructed from haplotypes that are too often from distant populations. One solution is to apply genomic control (Devlin and Roeder 1999) to the test-statistics in the case where inflation is observed but in general our method should only be applied in scenarios where an appropriately well-matched and large imputation panel is available. A final consideration is that our method assumes that the imputation panel represents the general population and hence does not include more case individuals (for the phenotype in question) than would be expected from a random population sample. Too many case individuals within the imputation panel, with similar haplotypes (potentially linked to the phenotypes), would likely also lead to a loss in power.

SURFBAT takes advantage of the highly efficient software IMPUTE5, and hence allows for huge imputation reference panels (such as the HRC, McCarthy *et al*. 2016 or TOPMED, Kowalski *et al*. 2019) to be used for association testing. As IMPUTE5 searches out the most appropriate haplotypes in the reference panel for each target haplotype, the presence of haplotypes in the reference panel that are not highly relevant to the imputation of a given target haplotype is not problematic. In this aspect, SURFBAT therefore resembles the UNICORN method (Bodea *et al*. 2016) where case individuals are compared with a large ensemble of reference individuals. In both the simulation study and real-data example presented here, performing a traditional GWAS adjusted for PCs was in fact more powerful than SURFBAT. However, in many cases such a strategy of directly performing a GWAS would not be possible and/or tractable as individual-level data for the largest imputation panels are not publicly available. However, SURFBAT is performed at the same time as imputation; opening up the possibility for online imputation servers such as the onein Michigan (Das *et al*. 2016) to also facilitate GWAS without having to share their data. This is demonstrated in the analysis of the Brugada dataset where SURFBAT is able to achieve similar results to a direct GWAS against the reference panel of NFE + FGR but without the requirement of jointly manipulating the case and control data together in order to calculate PCs and to perform the association testing. Even if individual-level data are available, for a huge control panel, merging the data with the case group, calculating PCs, and performing the GWAS can all be highly time consuming. There would nonetheless be an onus on the user of the imputation server to investigate whether the imputation panels available would likely be an appropriate control population for the purposes of the SURFBAT test, i.e. to establish whether the imputation panel would likely contain a sufficiently large number of individuals with ancestry profiles that span those present in the user's case group. Furthermore, the user would also have to verify that their association statistics were not largely inflated, which is always needed when performing genome-wide association testing with any method. Genomic control could be used in the case of inflation but would not guarantee that there would not remain associations resulting solely from population stratification. The onus would remain on the user to carefully interpret the association results and in the case of inflation to question whether the proximity of the study sample and imputation reference panel is such that the SURFBAT approach was reasonable.

Overall, the potential for using SURFBAT may not be very broad until a time when larger and more diverse (or indeed more specific) imputation reference panels become available on dedicated servers. There is also the additional restriction that GDPR regulations currently may prevent researchers from uploading their case sets onto imputation servers. However, progress on these fronts could be relatively swift. New imputation servers are becoming available, for example in Munich within the European Union (https://imputationserver.helmholtz-munich.de) (Rayner *et al*. 2024). The Genome of Europe (https://b1mg-project.eu/1mg/genome-europe) project will explore the possibility to develop a large set of population-specific imputation reference panels in coming years. There has also been significant recent research in the field of increasing data security on imputation servers (Dokmai *et al*. 2021; Kim *et al*. 2021; Sarkar *et al*. 2021). This may solve some regulatory issues and lead to the framework of an imputation server becoming more attractive for sharing and combining datasets for a range of different purposes beyond imputation.

It is instructive to zoom in on some of the Brugada association signals from Fig. 2 to better compare SURFBAT with a direct GWAS. In Supplementary Figs. 7–14, LocusZoom plots of the associations signals for SCN10A, HEY2,NCOA7, GATA4, and MYO18B are given for both SURFBAT and the direct GWAS against NFE + FGR adjusted for 6 PCs. The variants highlighted are the lead variants taken from Barc22. For SCN10A, we observe the similarity between the two strategies (Supplementary Figs. 7 and 8) and that SURFBAT had less power. Similar is also seen for MYO18B (Supplementary Figs. 13 and 14); here, there is clearly very little power in either strategy to detect the signal, but in particular for SURFBAT representing a key limitation of our method. Of course, the motivation for our method lies in scenarios where other approaches are impractical and in harnessing external control panels. For HEY2,NCOA7, again the two results are very similar (Supplementary Figs. 9 and 10). Both contain a peak that achieves genome-wide significance, again the GWAS has slightly higher power and interestingly SURFBAT has the lowest *P*-values for a set of variants with less strong linkage disequilibrium with the lead variant from Barc22. Finally, for GATA4 (Supplementary Figs. 11 and 12), neither strategy attains genome-wide significance though a suggestive peak is observable in Fig. 2. Interestingly, both methods give association peaks but with lead variants in only weak linkage disequilibrium with the lead variant of Barc22. For SURFBAT, the lead variant remains within GATA4 but for the GWAS it is actually in the neighboring gene FAM167A. This again shows that SURFBAT is a useful tool for exploring associations signals; providing an additional method, complementary to GWAS, to compare a case group to different sets of population-based controls.

Another key advantage of SURFBAT is that it only requires genotyping array data for case individuals, hence allowing for a very simple case-only design where instead of having to spread a sequencing budget across cases and controls, one could simply genotype a large number of case individuals and compare them to an appropriate existing imputation reference panel using SURFBAT. This could also prove advantageous when grouping case individuals together from many different cohorts. Indeed, when comparing SURFBAT to the FrEx GWAS study design, where both cases (Brugada) and controls (FrEx) are imputed, SURFBAT achieved genome-wide significance for the signal on HEY2,NCOA7 whereas the FrEx GWAS did not (Supplementary Fig. 4).

We have implemented the calculation of both paired and unpaired test-statistics in SURFBAT (see Methods). A comparison of the two tests is given in Supplementary Fig. 15 for the Brugada dataset example; the unpaired test attributed smaller *P*-values to the SNPs that are likely not following the null hypothesis near SCN10A on chromosome 3 and HEY2,NCOA7 on chromosome 6. However, the difference is very small, throughout this work it is the paired test-statistic from SURFBAT that has been presented.

The SURFBAT test could be readily extended for analysis on the X-chromosome, the only change that would have to be made would be to restrict the imputation reference panel to individuals carrying two X-chromosomes. This would be simple to do but would unfortunately lead to a loss in precision of the imputation and hence a poorer SURFBAT analysis as the size of the reference panel would likely be reduced by a factor of 2. This would also be a departure from the TDT parallel of SURFBAT, as it would ignore the fact that case individuals with two X-chromosomes would in fact only have one un-transmitted allele. It would potentially be feasible to first perform parent-of-origin analyses on the case individuals and hence construct a test-statistic in the spirit of a true chromosome-X TDT analyses (Ho and Bailey-Wilson 2000), which would focus only on transmission on the maternal side. However, this would likely be a very impractical study design.

Continuous phenotypes could also be readily analyzed using both the imputed dosages and imputed “pseudo-control” dosages provided by SURFBAT. One possible way that this could be done would be to use the “sib-GWAS” regression models of Howe *et al*. (2022). The idea would be to regress the phenotypes of the study individuals against their (imputed) genotypes with a controlling covariable of the approximate mean parental genotypes that would be constructed as the average (for each study individual) of their imputed allele dosages and their paired “pseudo-controls.” The application of SURFBAT in IMPUTE5 stores the four haplotype dosages (two imputed and two “un-transmitted” for the “pseudo-control”) per individual. With these four dosages, and depending on the context of the study sample, there is hence the possibility to apply other statistical approaches derived for family based data; notably including those that generalize the TDT framework for quantitative traits (Spielman and Ewens 1998; Abecasis *et al*. 2000; Horvath *et al*. 2001; Shih and Whittemore 2002; Cobat *et al*. 2014).

SURFBAT was developed with a particular scenario in mind, where the case individuals have only genotyping data. However, the same methodology could eventually be applied when the case individuals have either low- or high-coverage sequencing data. Exome data would likely not work as performing imputation on to exome data usually gives very poor results as a certain density of frequent markers in the target data is required. We also had in mind a scenario where the case individuals are possibly admixed while the imputation reference panel is not. This is the case when recruitment of controls is based on geographical criteria such as is the case with the FGR panel and so the haplotypes within the reference individuals are ancestry-matched by design. The conditions of the test mean that it should only be applicable to study designs involving such reference panels characteristics. Nevertheless, for increasingly large reference panels such as TOPMED or Genomics England (Shi *et al*. 2024) that can be freely accessed for imputation studies on imputation servers, the majority of reference individuals will not be recently admixed and so haplotypes within individuals will likely be approximately matched on ancestry in a broad sense. Hence, the SURFBAT test should still work relatively well, though we would recommend some caution in interpreting the results and that inflation in the test-statistics might occur.

## Conclusion

SURFBAT provides an interesting counterpoint to GWAS and is very practical to put in place as we have constructed the test so that it is achieved simultaneously to the imputation of genotypes with IMPUTE5. As imputation reference panels have become incredibly large, SURFBAT provides a methodology to harness such panels for association testing efficiently. It must however be stressed that the performance of SURFBAT therefore depends entirely on the availability of an appropriate imputation reference panel for one’s set of case individuals. A key limitation of our method to signal was that SURFBAT was less powerful than GWAS in both the simulations and real-data example. This diminished power results from the fact that we are essentially comparing K cases with K pseudo-controls. When we performed our GWAS of Brugada, we were comparing 356 cases to the entire imputation reference panel and so 1,260 controls. Hence, while the SURFBAT test may have benefitted from a more precise model for local ancestry, any gain in power was nullified when compared with the GWAS with a much higher sample size for the controls. As discussed, a fairer comparison might be against the GWAS involving FrEx, which included 346 cases and 569 controls. Here, SURFBAT had much more comparable power and indeed a smaller lead *P*-value for the previously observed signal on HEY2,NCOA7.

## Data and software availability

SURFBAT is available as a functionality in the existing software IMPUTE5 (https://jmarchini.org/software/). Simulation scripts and materials are available at https://github.com/a-herzig/surfbat. This study generated no new data, and relies of datasets described in previous articles. The key datasets used were FGR and Brugada, which are described in Alves *et al*. (2024) and Barc *et al*. (2022), respectively. A large subset of the FGR dataset is available on the EGA (EGAD00010002661) and full instructions for data access can be found in (Alves *et al*. 2024). WGS data from Alves *et al*. (2024) and Barc *et al*. (2022) will be submitted to the French Centralized Data Center of the France Medicine Genomic Plan that is currently under construction (https://pfmg2025.aviesan.fr/le-plan/collecteur-analyseur-de-donnees-cad/). Researchers must submit a research proposal to access the data and to gain a remote access to the CAD server; in compliance with French legislation on identifiable personal data and its compatibility with the legislation of the requesting country. Researchers may need to complete a full application for the re-utilization of the data, agree to re-contact the participants and eventually to remove participants not consenting to the re-analysis of their data. An application to the French National Commission on Informatics and Liberty may be required depending on the nature of the new analyses proposed. Requests for use of these data for research in population genetics should be initially directed to the LABEX GENMED (http://www.genmed.fr/index.php/en/contact). To access individual data, applicants should submit a synopsis of their project to the FrenchGenRef Data Access Committee composed of the three Principal Investigators: Emmanuelle Génin (emmanuelle.genin@inserm.fr), Richard Redon (richard.redon@univ-nantes.fr), and Jean-François Deleuze (deleuze@cnrgh.fr). Those wishing to access the Brugada data on a collaborative basis should contact Julien Barc (julien.barc@inserm.fr) and Richard Redon (richard.redon@inserm.fr). Aggregated data for the FrEx dataset is available at http://lysine.univ-brest.fr/FrExAC/, those wishing to access the data on a collaborative basis should contact Emmanuelle Génin (emmanuelle.genin@inserm.fr). Summary statistics for the results of SURFBAT and the GWAS for the Brugada analyses are also available at https://github.com/a-herzig/surfbat and at GSA FigShare https://doi.org/10.25387/g3.27901218.

Supplemental material available at G3 online.

## Supplementary Material

jkae287_Supplementary_Data

## Consortium information

**The FGR Consortium**

Principal Investigators

Jean-François Deleuze^5,6^

Emmanuelle Génin^1,9^

Richard Redon^7^

Collaborators

Data collection: Chantal Adjou^6^, Stéphanie Chatel^7^, Claude Férec^1,9^, Marcel Goldberg^11^, Philippe-Antoine Halbout^10^, Hervé Le Marec^7^, David L’Helgouach^10^, Karen Rouault^1,9^, Jean-Jacques Schott^7^, Anne Vogelsperger^10^, and Marie Zins.^11^

Sample preparation/sequencing: Delphine Bacq^5^, Hélène Blanché^6^, Anne Boland^5^, Robert Olaso.^5^

Bioinformatics: Pierre Lindenbaum^7^, Thomas Ludwig^1,9^, Vincent Meyer^5^, Florian Sandron^5^, Damien Delafoy^5^, and Lourdes Velo-Suárez.^1,9^

Statistical Genetics: Isabel Alves^7^, Ozvan Bocher^1^, Christian Dina^7^, Anthony F. Herzig^1^, Matilde Karakachoff^7^, Gaëlle Marenne^1^, Aude Saint Pierre^1^, and Véronique Geoffroy.^1^

1. Univ Brest, Inserm, EFS, UMR 1078, GGB, F-29200 Brest, France

5. Université Paris-Saclay, CEA, Centre National de Recherche en Génomique Humaine (CNRGH), F-91000 Evry, France

6. Fondation Jean Dausset—Centre d’Etude du Polymorphisme Humain (CEPH), F-75010 Paris, France

7. Nantes Université, CHU Nantes, CNRS, INSERM, l’institut du thorax, F-44000 Nantes, France

9. CHRU Brest, F-29200 Brest, France

10. Etablissement Français du Sang, F-94210 La Plaine Saint-Denis, France

11. UMS 11, Inserm, Université de Versailles, F-78885 Saint-Quentin-en-Yvelines, France

**The FrEx Consortium**

Principal Investigators

Emmanuelle Génin^1,9^ (chair)

Dominique Campion^12^

Jean-François Dartigues^15^

Jean-François Deleuze^5,6^

Jean-Charles Lambert^14^

Richard Redon^7^

Collaborators

Bioinformatics: Thomas Ludwig^1,9^ (chair), Benjamin Grenier-Boley^14^, Sébastien Letort^1,9^, Pierre Lindenbaum^7^, Vincent Meyer^5^, Olivier Quenez^12^

Statistical genetics: Christian Dina^7^ (chair), Céline Bellenguez^14^, Camille Charbonnier-Le Clézio^12^, Joanna Giemza^7^

Data collection: Stéphanie Chatel^7^, Claude Férec^1,9^, Hervé Le Marec^7^, Luc Letenneur^11^, Gaël Nicolas^12^, Karen Rouault^1,9^

Sequencing: Delphine Bacq^5^, Anne Boland^5^, Doris Lechner^5^

1. Univ Brest, Inserm, EFS, UMR 1078, GGB, F-29200 Brest, France

5. Université Paris-Saclay, CEA, CNRGH, F-91000 Evry, France

6. Fondation Jean Dausset—CEPH, F-75010 Paris, France

7. Nantes Université, CHU Nantes, CNRS, INSERM, l’institut du thorax, F-44000 Nantes, France

9. CHRU Brest, F-29200 Brest, France

12. Univ Rouen, Inserm UMR 1079, F-76000 Rouen, France

13. Univ Bordeaux, Inserm UMR 1219, F-33000 Bordeaux, France

14. Inserm UMR 1167, Institut Pasteur, F-59000 Lille, France
