# Supplementary material for: SURFBAT: a surrogate family based association test building on large imputation reference panels
Source: G3 (Bethesda). 2024 Dec 9;15(4):jkae287. doi: 10.1093/g3journal/jkae287 (PMC12005154; doi:10.1093/g3journal/jkae287)
Supplement: jkae287_Supplementary_Data [file jkae287_supplementary_data.docx]

**Supplementary Materials**

**SURFBAT: a surrogate family-based association test building on large imputation reference panels**

AF Herzig^1,*^, S Rubinacci^2^, G Marenne^1^, H Perdry^3^, FrEx Consortium^1^, FranceGenRef Consortium^4^, J-F Deleuze^5,6^, C Dina^7^, J Barc^7^, R Redon^7^, O Delaneau^8^, E Génin^1,9^

1: Inserm, Univ Brest, EFS, UMR 1078, GGB, F-29200 Brest, France

2: Institute for Molecular Medicine Finland, University of Helsinki, 00290 Helsinki, Finland

3: CESP Inserm U1018, Université Paris-Saclay, F-94807 Villejuif, France

4: LABEX GENMED, Centre National de Recherche en Génomique Humaine, F-91000 Evry, Paris, France

5 : Université Paris-Saclay, CEA, Centre National de Recherche en Génomique Humaine (CNRGH), F-91000 Evry, France

6: CEPH, Fondation Jean Dausset, F-75010 Paris, France

7: Nantes Université, CNRS, INSERM UMR 1087, l’institut du thorax, F-44000 Nantes, France

8: Regeneron Genetics Center, Tarrytown, NY 10591, USA

9: CHU Brest, F-29200 Brest, France

* Corresponding Author ([anthony.herzig@inserm.fr](mailto:anthony.herzig@inserm.fr), IBRBS INSERM UMR1078, 22 Avenue Camille Desmoulins, 29238 Brest, France)

| SNP | Gene | Barc et al, 2022(Barc et al. 2022) | GWAS with 6 PCs  against NFE+FGR | SURFBAT  against NFE+FGR | GWAS  with 6 PCs against FrEx  using NFE+FGR for imputation | GWAS with 6 PCs  against 1000G+FGR | SURFBAT  against 1000G+FGR | SURFBAT unpaired against NFE+FGR |
| --- | --- | --- | --- | --- | --- | --- | --- | --- |
| rs7638909 | SCN5A | **2.79e-8** | **8.98e-5** | **1.12e-4** | **1.06e-3** | **1.22e-4** | **3.61e-3** | **5.68e-5** |
| rs62241190 | SCN5A | **8.56e-14** | **3.44e-3** | **2.41e-3** | **0.016** | **1.77e-3** | **9.87e-4** | **2.09e-3** |
| rs7374540 | SCN5A | **3.56e-57** | **1.03e-7** | **4.56e-6** | **9.52e-5** | **1.14e-7** | **3.72e-4** | **3.25e-6** |
| rs7433206 | SCN5A | **9.52e-24** | 0.280 | 0.967 | 0.168 | 0.295 | 0.253 | 0.967 |
| rs34760424 | SCN5A | **3.03e-23** | **1.86e-4** | **7.45e-3** | **1.44e-4** | **2.53e-4** | **0.018** | **5.07e-3** |
| rs41310232 | SCN5A | **1.19e-15** | **1.28e-5** | **1.17e-4** | **5.88e-3** | **4.37e-6** | **7.86e-5** | **5.84e-5** |
| rs6782237 | SCN5A | **1.05e-47** | **2.15e-8** | **1.40e-7** | **2.65e-6** | **1.42e-8** | **3.36e-7** | **1.00e-7** |
| rs6801957 | SCN10A | **1.30e-180** | **1.05e-26** | **3.23e-17** | **5.29e-21** | **1.38e-26** | **2.68e-18** | **4.33e-20** |
| rs6913204 | HDDC2 | **1.3e-8** | 0.290 | 0.559 | 0.210 | 0.359 | 0.167 | 0.577 |
| rs9398791 | HEY2,NCOA7 | **1.49e-39** | **3.03e-9** | **1.11e-7** | **1.51e-6** | **1.20e-9** | **2.92e-9** | **3.40e-8** |
| rs11765936 | TBX20 | **4.3e-11** | **4.25e-4** | **2.24e-4** | **9.92e-3** | **9.90e-4** | **1.93e-4** | **1.50e-4** |
| rs340398 | TBX20 | **1.76e-9** | 0.250 | 0.945 | **0.031** | 0.240 | 0.467 | 0.945 |
| rs804281 | GATA4 | **1.22e-9** | **5.54e-4** | **0.041** | 0.093 | **6.81e-4** | 0.645 | **0.043** |
| rs72671655 | ZFPM2 | **2.51e-13** | **2.84e-3** | **1.93e-3** | **2.44e-3** | **1.07e-3** | **4.79e-3** | **1.04e-3** |
| rs72905083 | WT1 | **2.09e-9** | **0.035** | **0.032** | 0.192 | **0.042** | **9.09e-3** | **0.035** |
| rs883079 | TBX5 | **1.59e-10** | **0.020** | **0.017** | 0.063 | **0.011** | 0.361 | **0.015** |
| rs11645463 | IRX3 | **1.27e-9** | 0.100 | 0.367 | 0.181 | 0.065 | 0.243 | 0.357 |
| rs72622262 | CRNDE,IRX5 | **1.37e-11** | **0.030** | 0.171 | 0.151 | 0.061 | **0.012** | 0.180 |
| rs12945884 | PRKCA | **3.31e-8** | **0.027** | 0.302 | **0.013** | **0.023** | 0.364 | 0.280 |
| rs476348 | MAPRE2 | **2.64e-9** | **0.014** | 0.480 | **4.96e-4** | **0.016** | 0.845 | 0.479 |
| rs133902 | MYO18B | **7.73e-9** | **2.29e-3** | **0.024** | **2.59e-3** | **2.37e-3** | 0.226 | **0.022** |

Supplementary Table 1: P-values from the association analyses presented in Figure 2 in the main text and Supplementary Figures 3 and 4 for the set of SNPs identified in Table 1 of (Barc et al. 2022). We highlight in gold the p-values below 5e-8 and in blue those below 0.05.


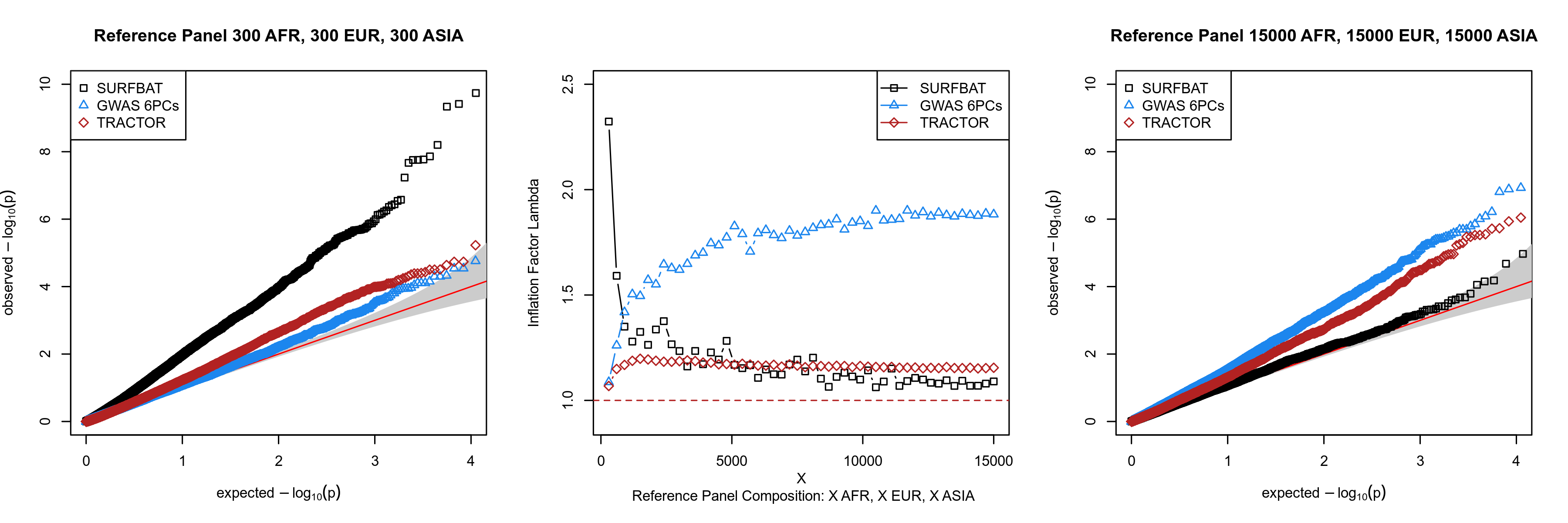


Supplementary Figure 1: Using the msprime (Baumdicker et al. 2022) simulation described in the main text, we applied the SURFBAT test to a group of 2000 admixed individuals against reference panels containing X individuals from AFR, EUR and ASIA respectively; giving a reference panel of total size 3X where X ranges from 300 to 15,000 and hence the total reference panel size ranges from 900 to 45,000. A genome-wide association study adjusted for 6 Principal Components (GWAS 6PCs) and TRACTOR (Atkinson et al. 2021) were also performed. We report the inflation factor $\lambda$ for each different method and for the different sized reference panels (central plot). Demonstrative quantile-quantile plots (qq-plots) for the most extreme iterations (X=300 and X=15,000) are given in the left and right plots respectively.


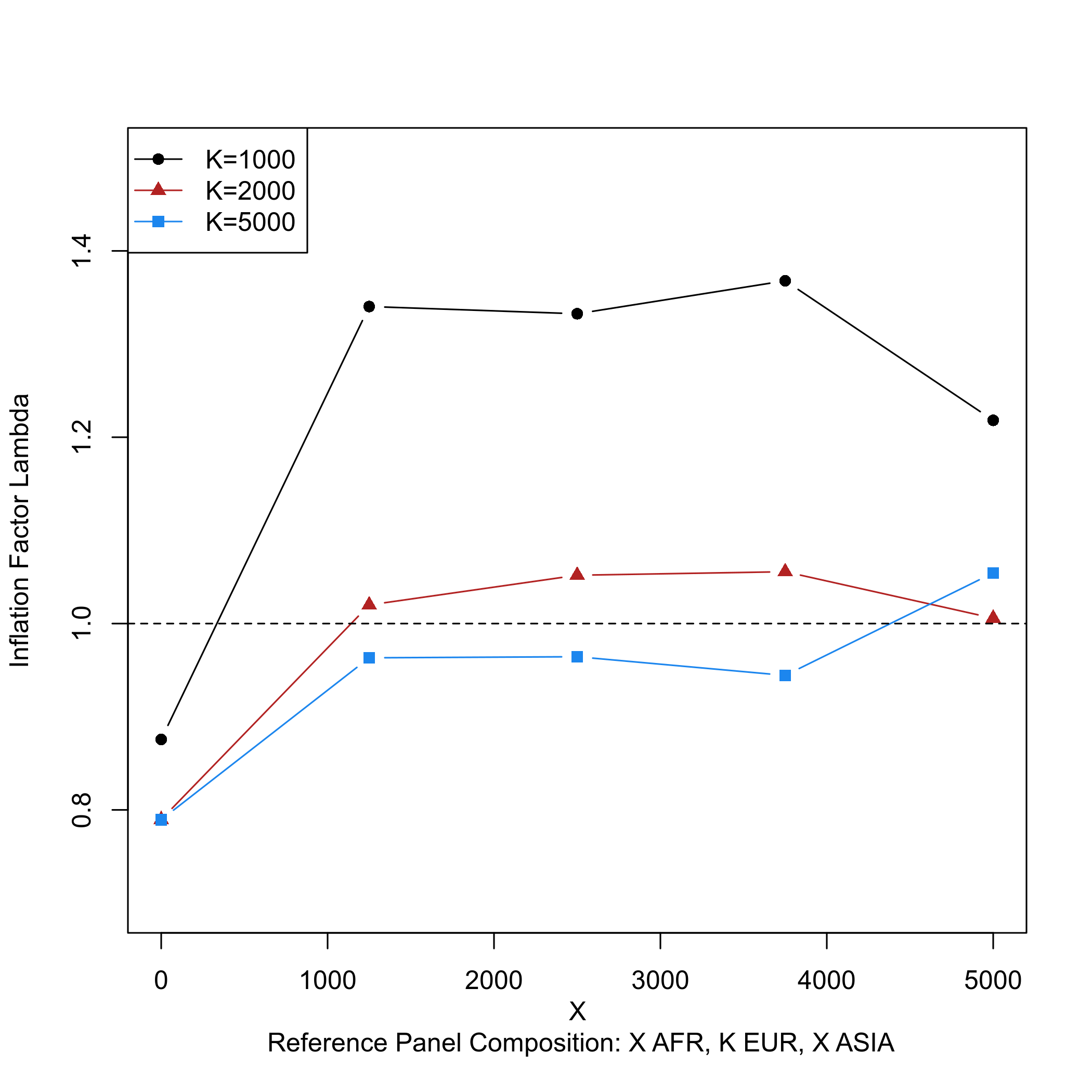


Supplementary Figure 2: Again using the msprime simulation described in the main text, 500 individuals were created as mosaics of the 15,000 simulated individuals with European ancestry and SURFBAT was applied using reference panels that contained either K=1,000, 2,000, or 5,000 individuals with simulated European ancestry and X individuals with simulated African and Asian ancestry for values of X that range between 0 and 5,000. The resultant inflation factor $\lambda$ of the SURFBAT test statistics are compared for different values of X and K.


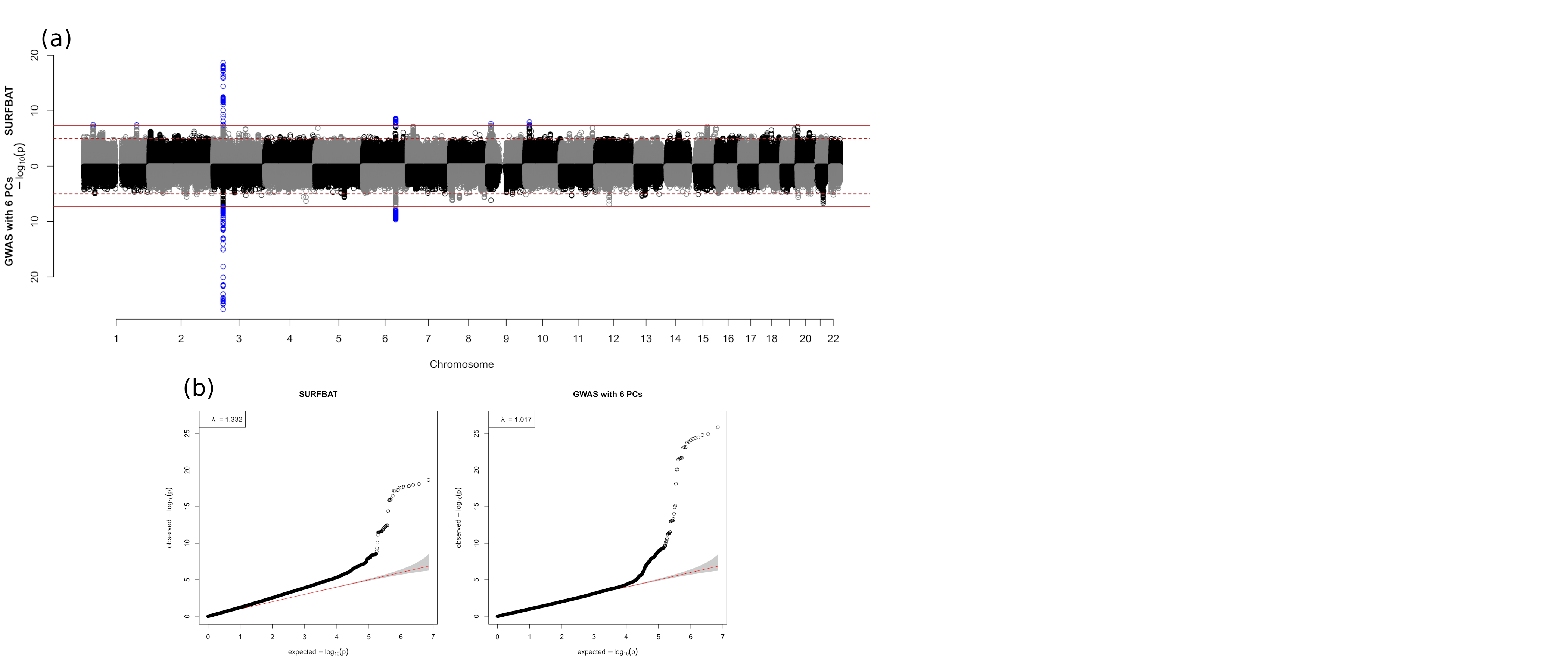


Supplementary Figure 3: Equivalent to Figure 2 in the main text but here in place of the NFE+FGR reference panel (containing 404 individuals of non-Finnish European Ancestry from the 1000 Genomes Project (1000G) [4] and 856 individuals from the FranceGenRef (FGR) cohort [5,6]), the whole of the 1000G was combined with FGR (1000G+FGR reference panel).


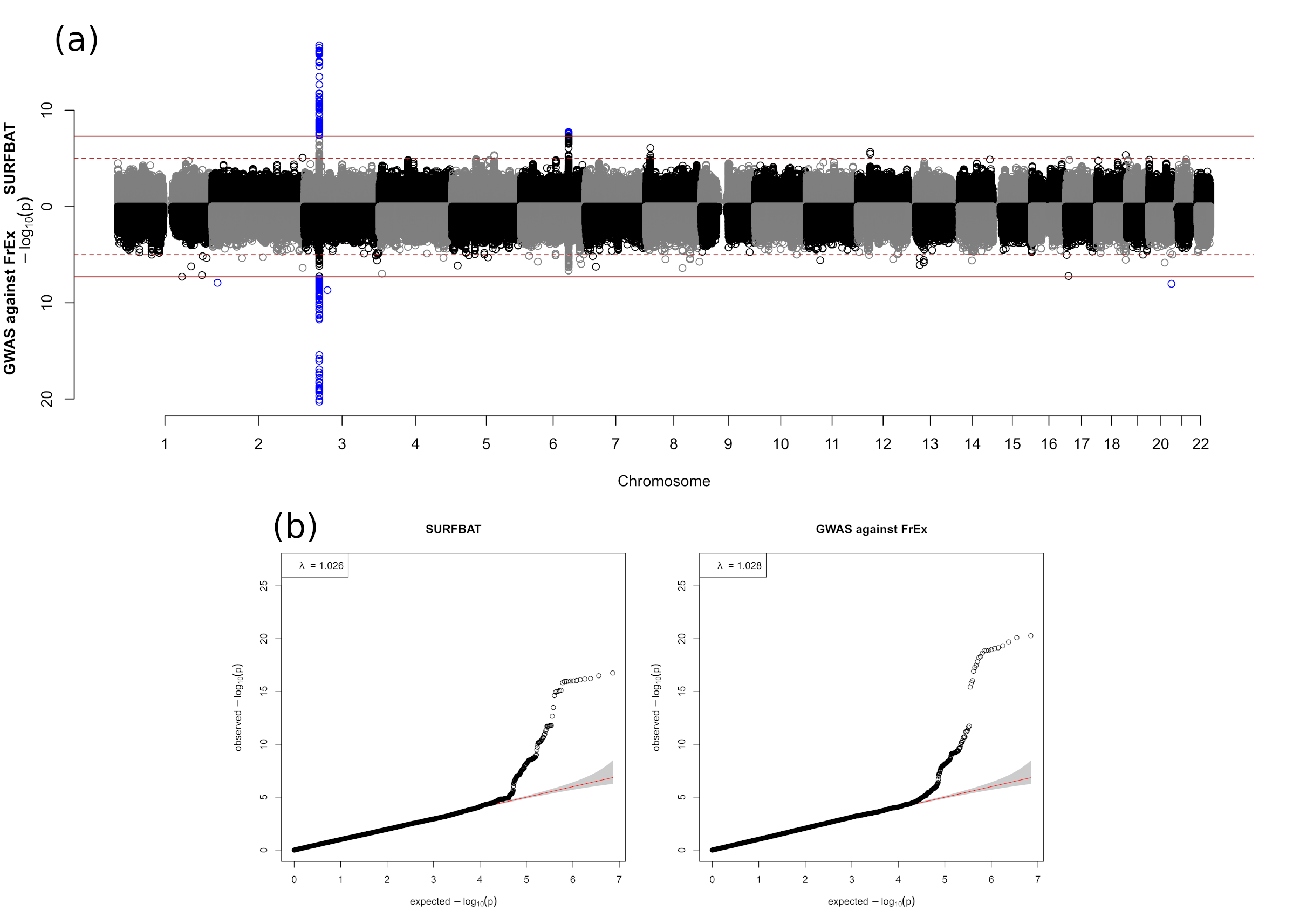


Supplementary Figure 4: Equivalent to Figure 2 in the main text but here SURFBAT is compared to the FrEx GWAS strategy.


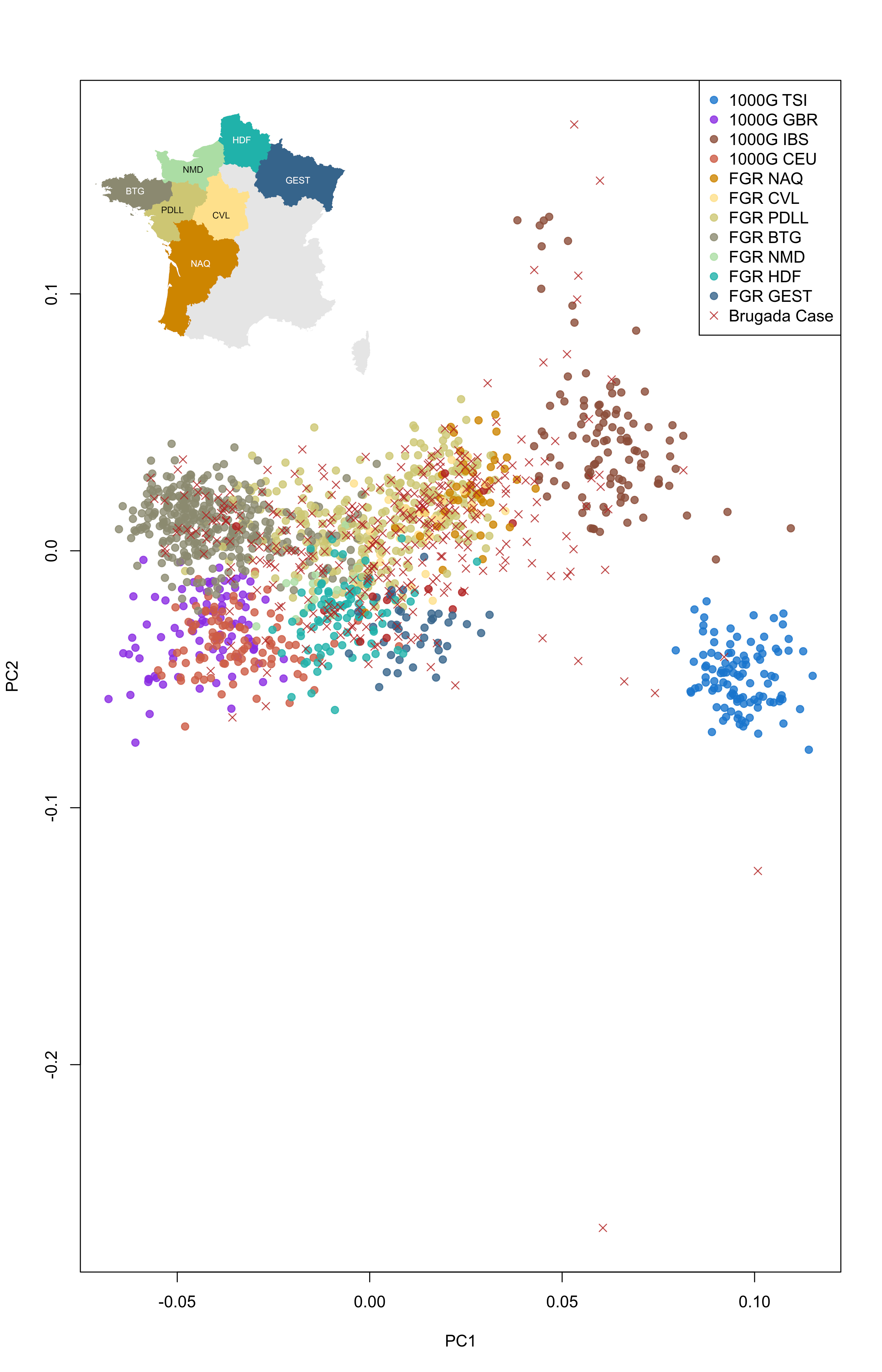


Supplementary Figure 5: Full principal components analysis of the case and control individuals pertaining to the analysis of Figure 2 in the main text.


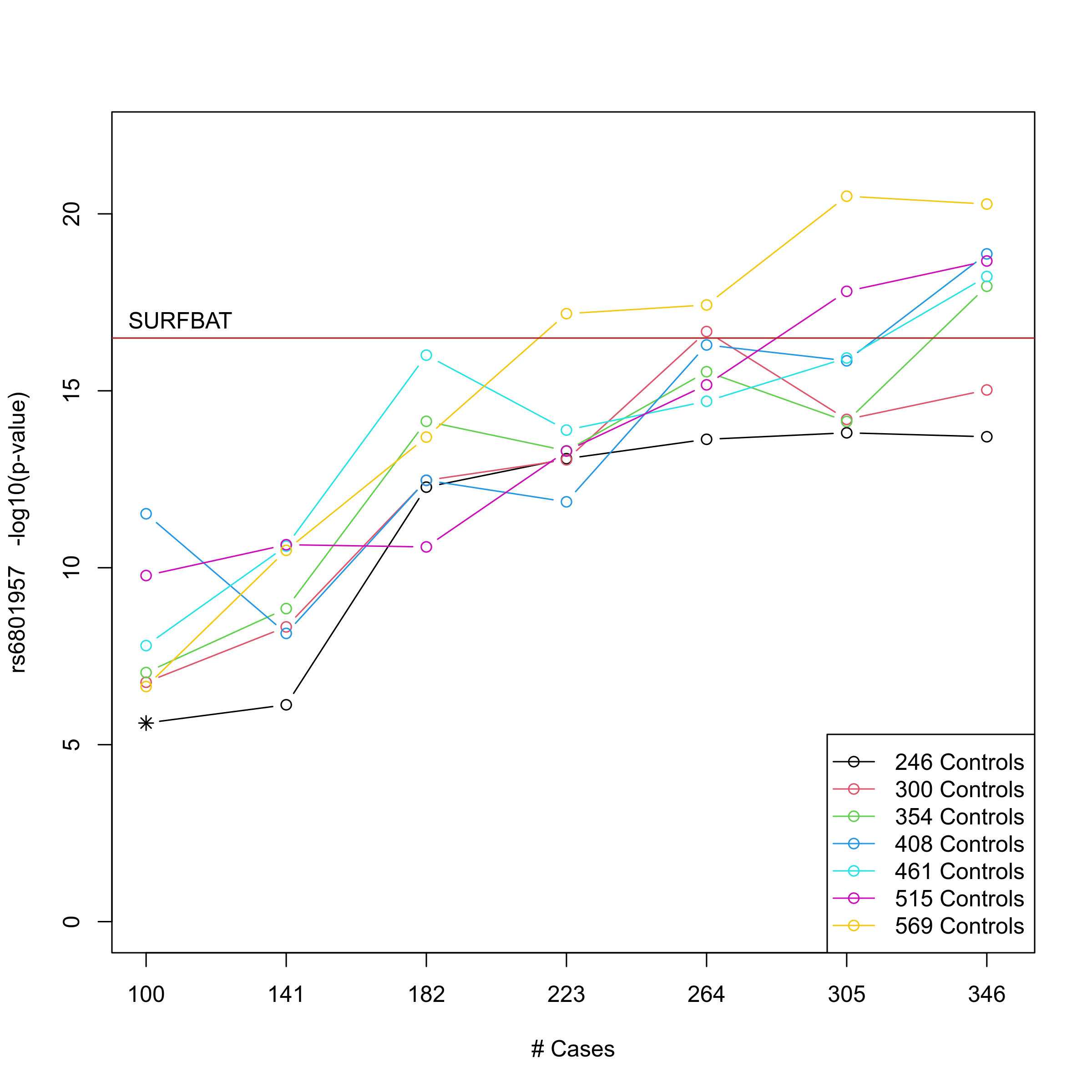


Supplementary Figure 6: In the ‘GWAS against FrEx’ strategy, we varied the number of cases (x-axis) and number of controls (colours) via random down-sampling. We report here the p-value of rs6801957, the lead SNP in the SCN10A GWAS peak. A horizontal line in red represents the p-value that was afforded by the SURFBAT test using all 346 cases (Figure 2 in main text, Supplementary Table 1). The increased power with increased samples sizes of cases and controls is clearly observed, and we also observe how the power of SURFBAT is indeed similar to a scenario involving the same number of cases and controls (in this case 346). The first point on the black line is denoted with a star as this is the only scenario where the same number of total individuals (346) would have to be genotyped for the ‘GWAS against FrEx’ strategy as would be needed for the SURFBAT strategy.


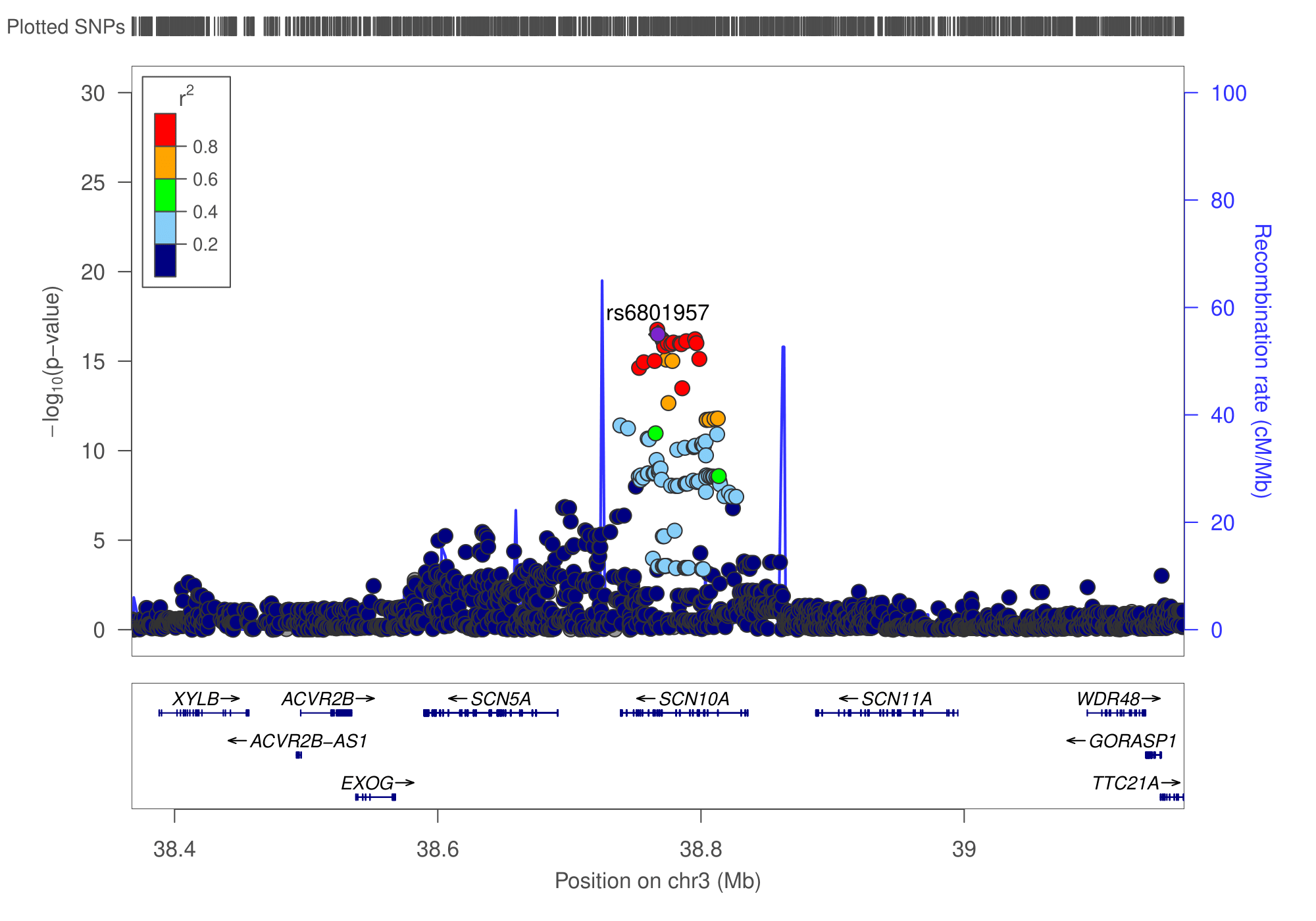


Supplementary Figure 7: Locus Zoom on *SCN10A* for the SURFBAT strategy presented in Figure 2 in the main text.


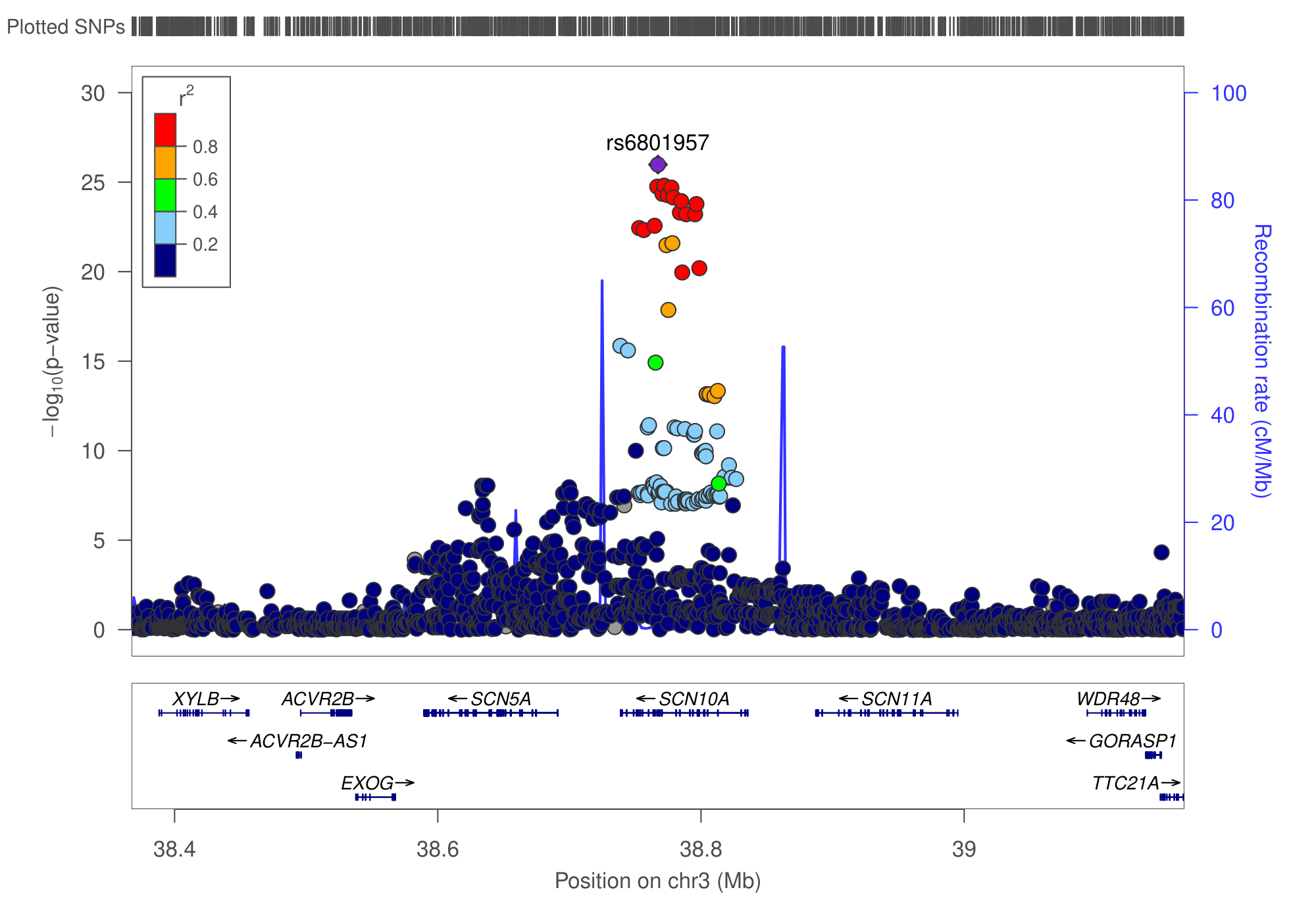


Supplementary Figure 8: Locus Zoom on *SCN10A* for the GWAS + 6 PCs strategy presented in Figure 2 in the main text.


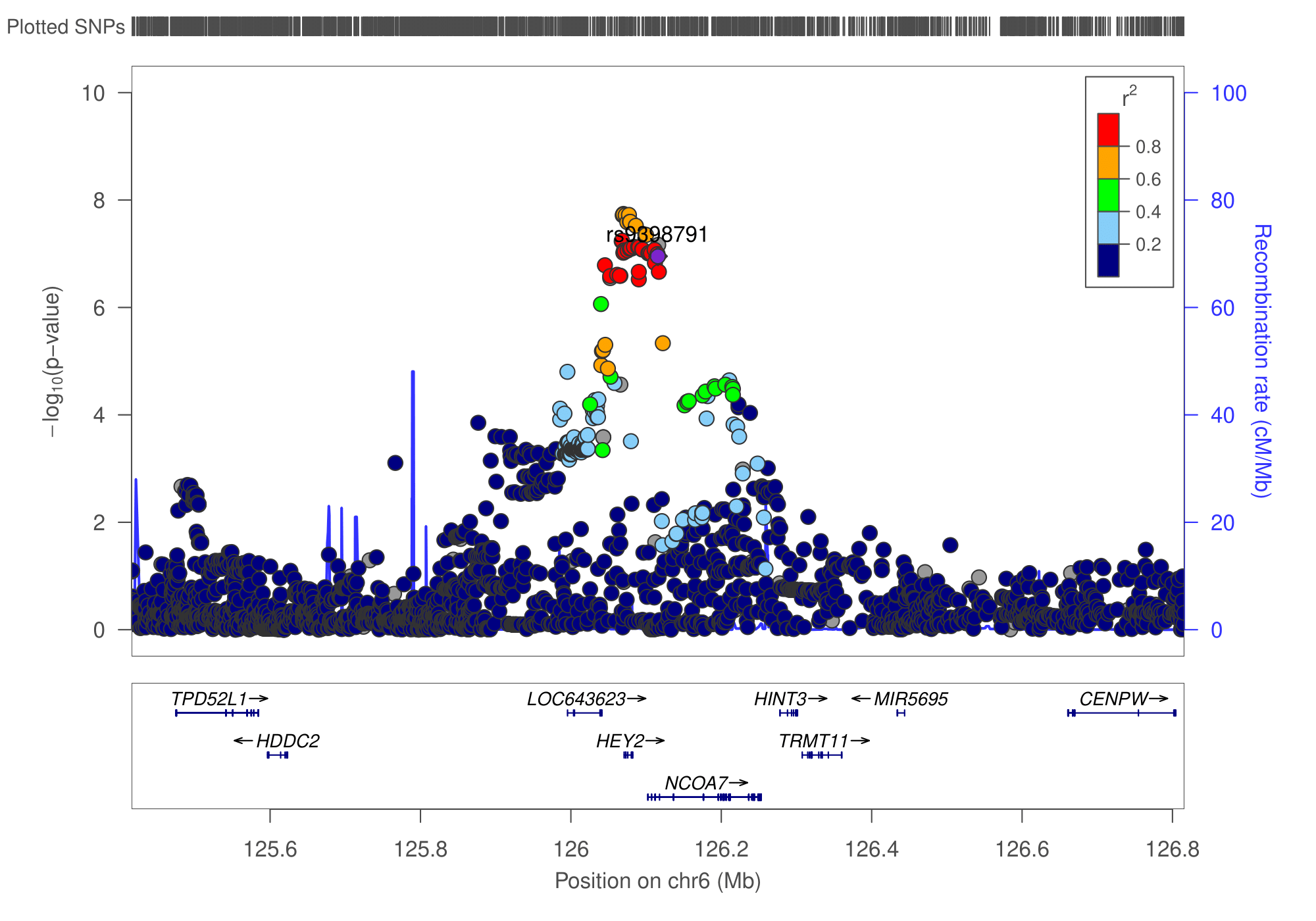


Supplementary Figure 9: Locus Zoom on *HEY2,NCOA7* for the SURFBAT strategy presented in Figure 2 in the main text.


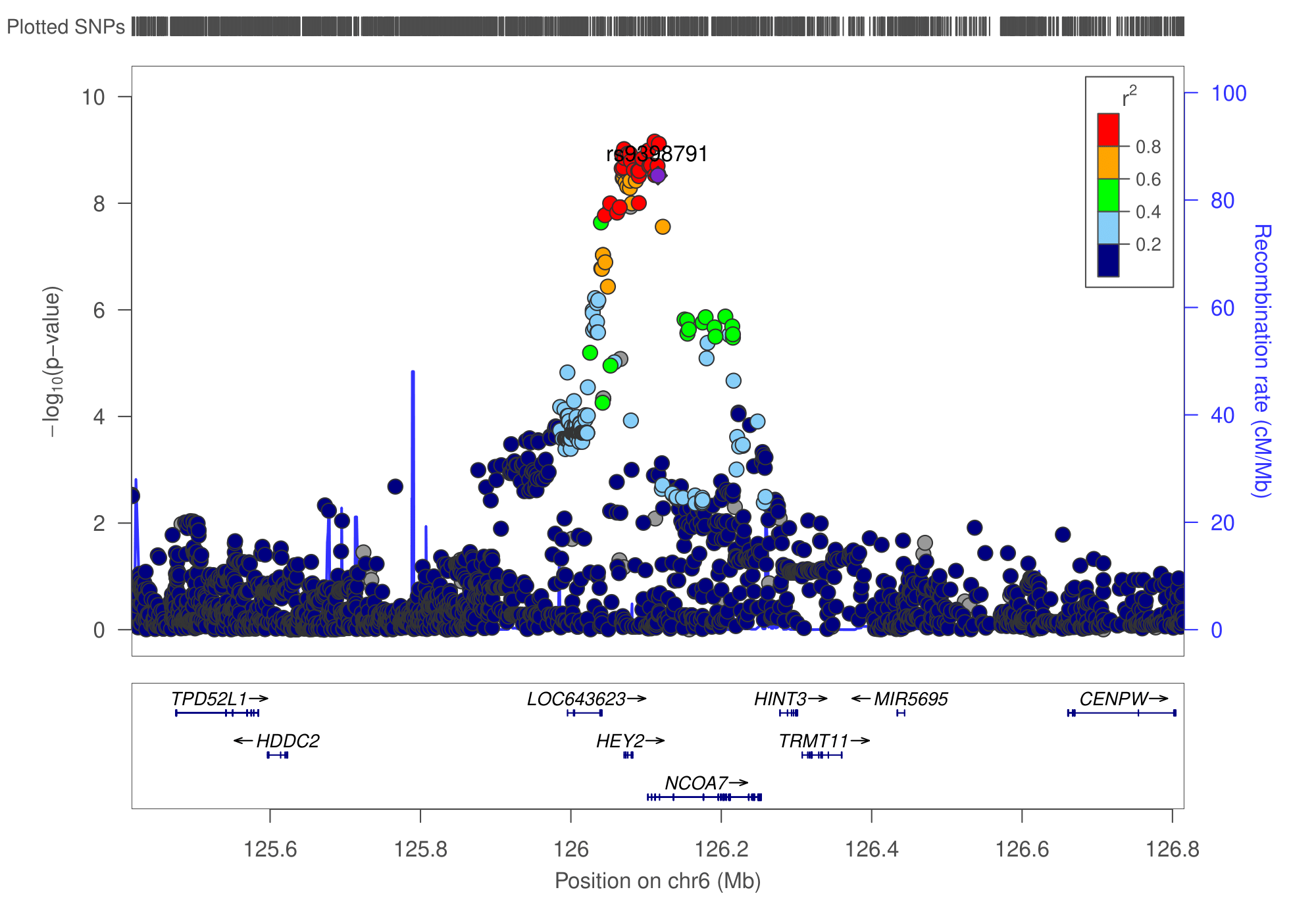


Supplementary Figure 10: Locus Zoom on *HEY2,NCOA7* for the GWAS + 6 PCs strategy presented in Figure 2 in the main text.


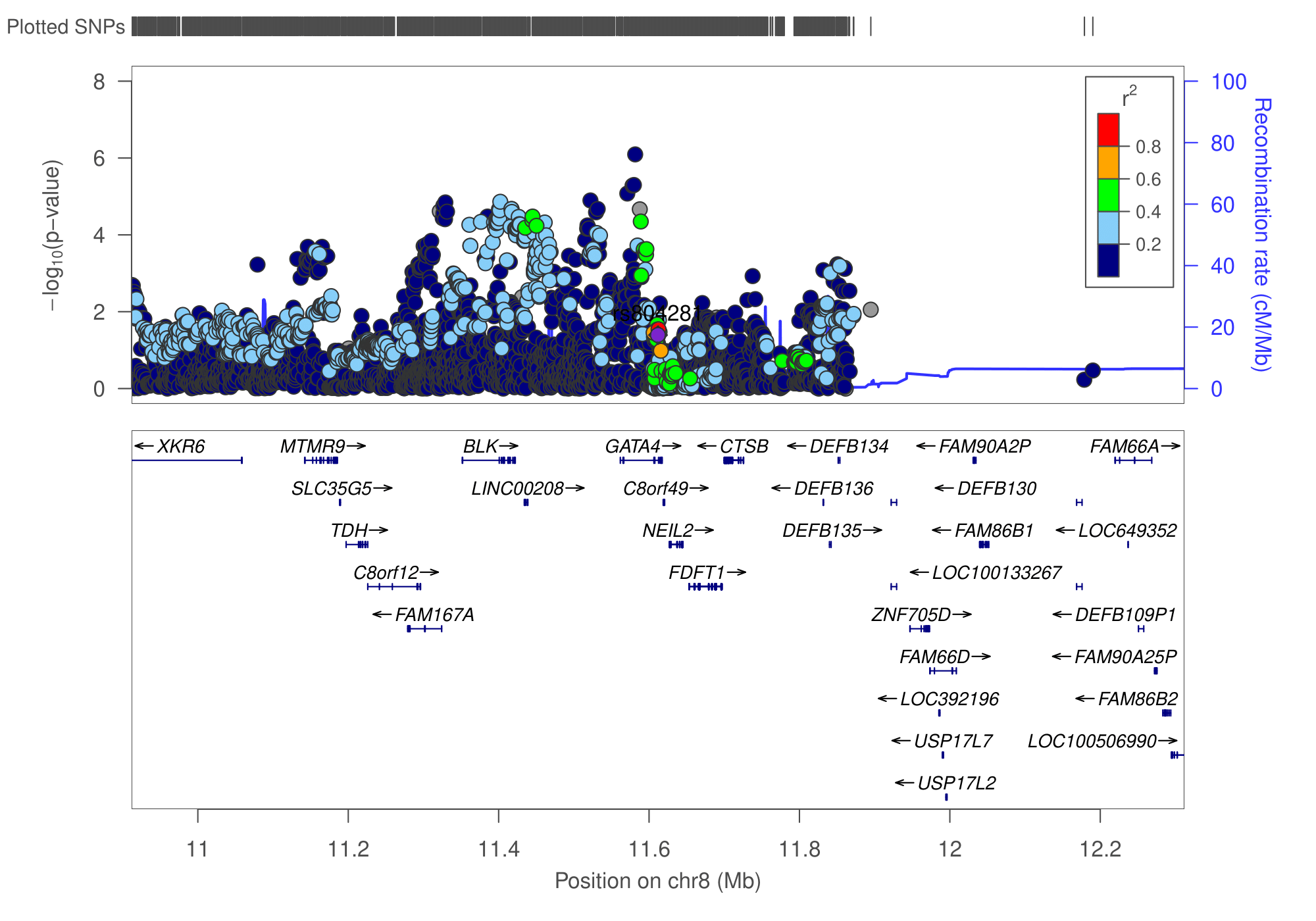


Supplementary Figure 11: Locus Zoom on *GATA4* for the SURFBAT strategy presented in Figure 2 in the main text.


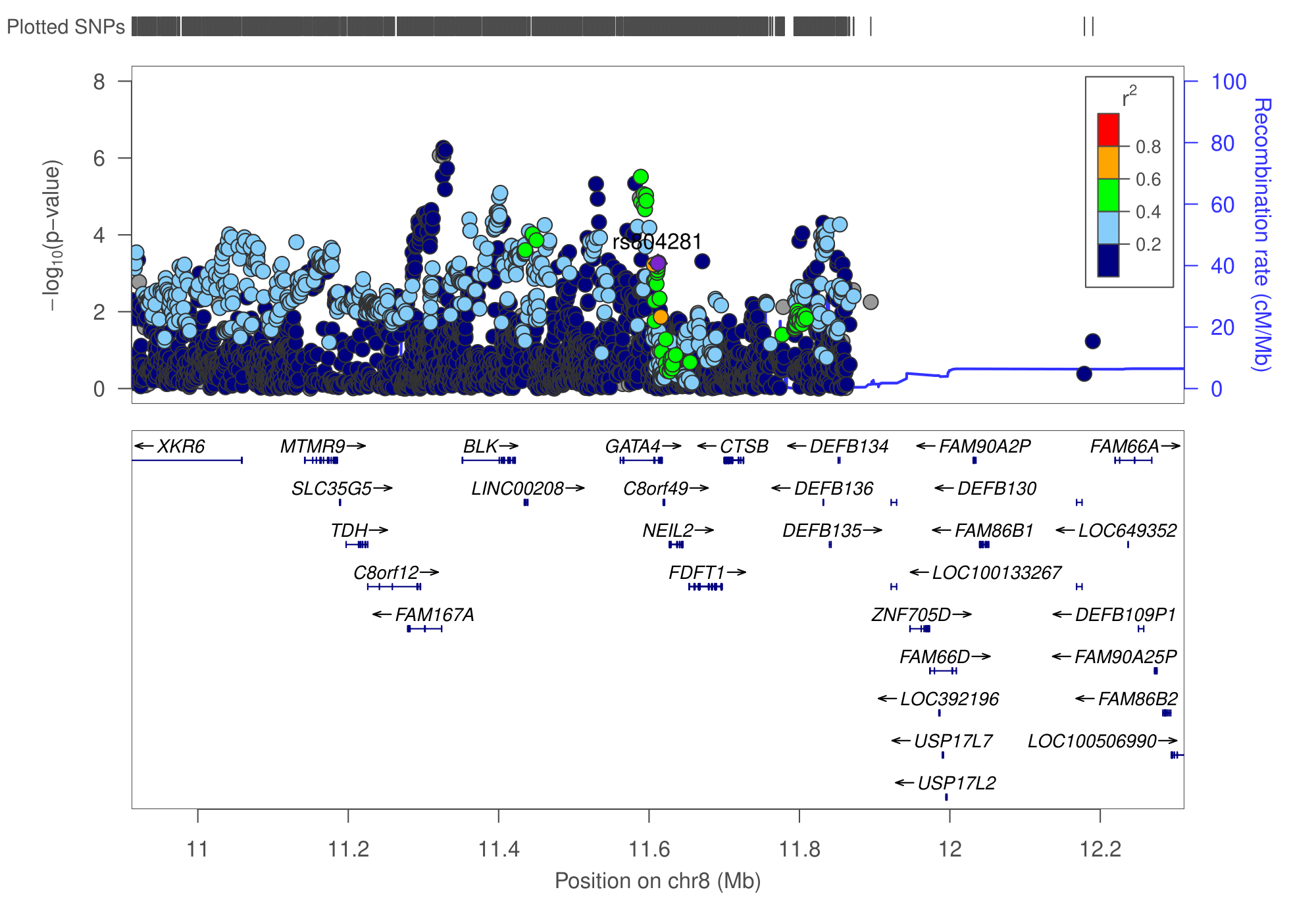


Supplementary Figure 12: Locus Zoom on *GATA4* for the GWAS + 6 PCs strategy presented in Figure 2 in the main text.


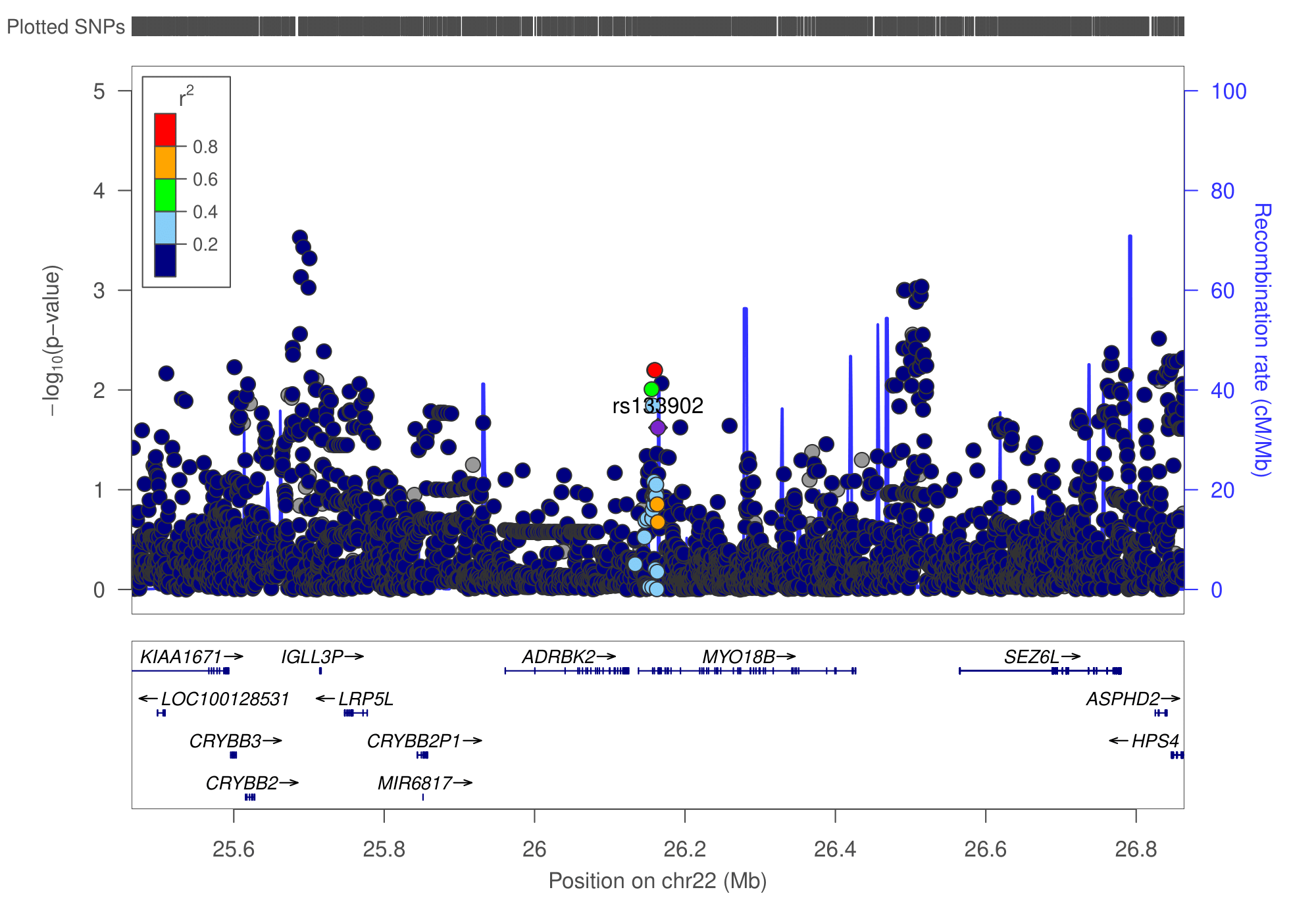


Supplementary Figure 13: Locus Zoom on *MYO18B* for the SURFBAT strategy presented in Figure 2 in the main text.


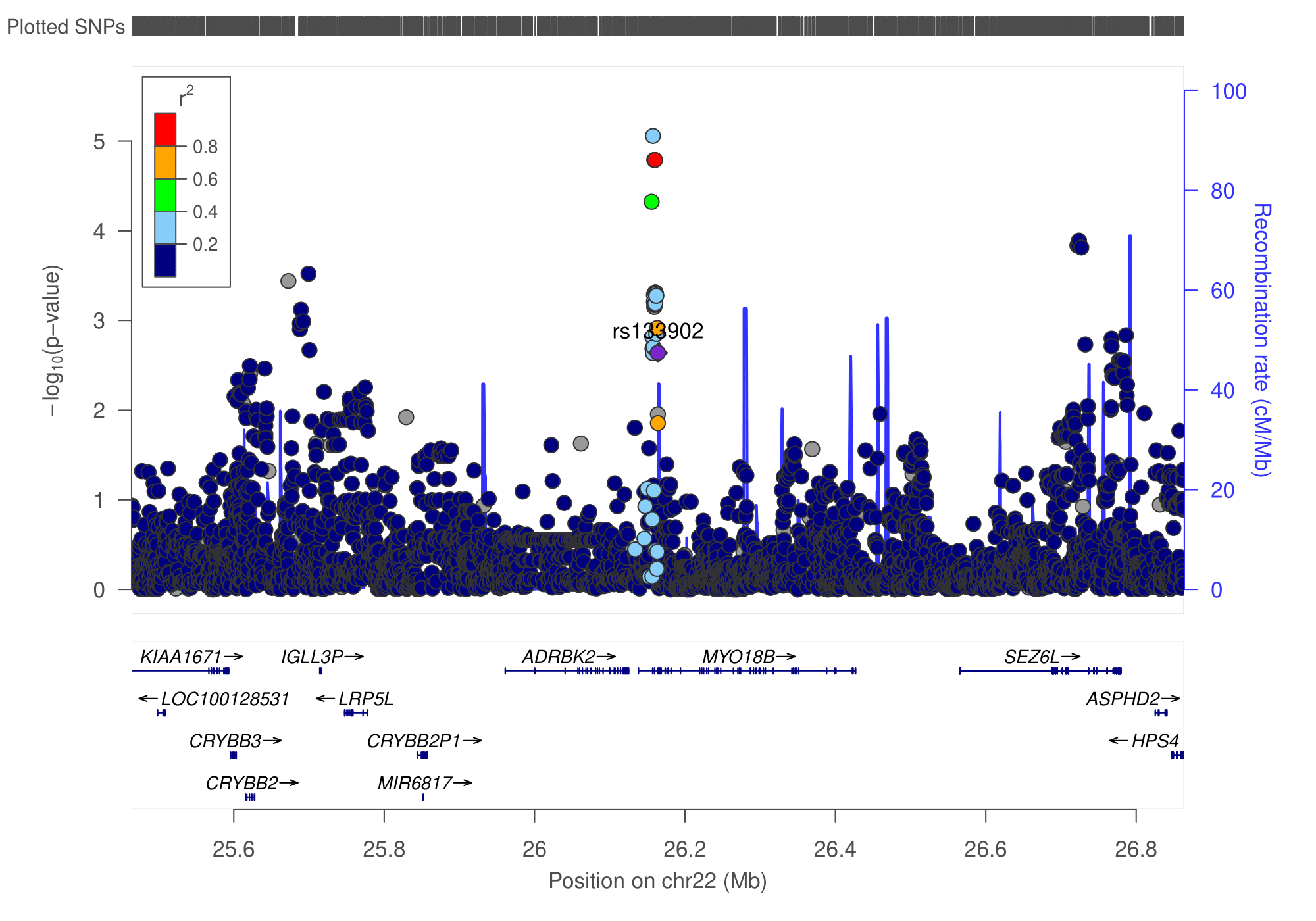


Supplementary Figure 14: Locus Zoom on *MYO18B* for the GWAS + 6 PCs strategy presented in Figure 2 in the main text.


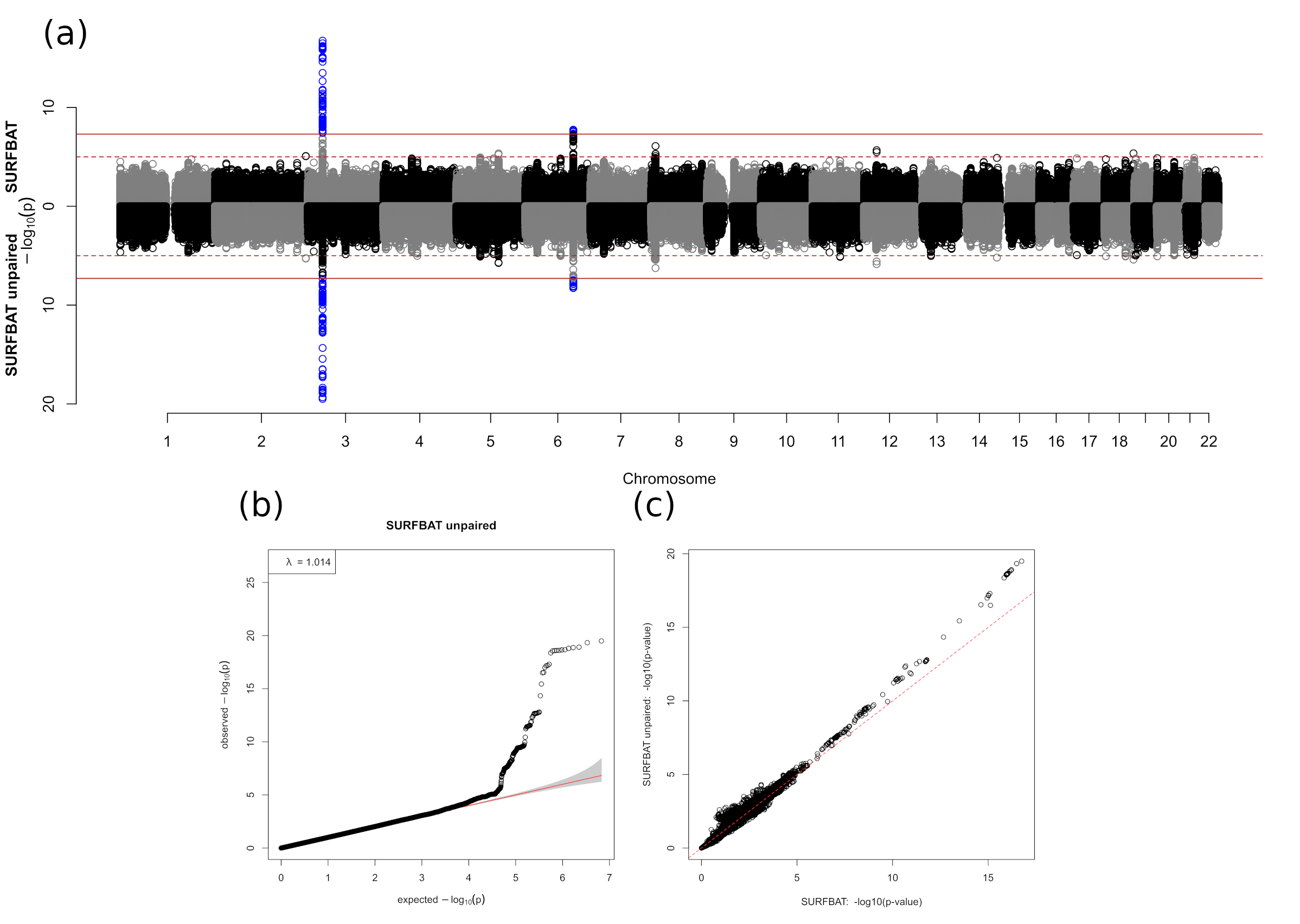


Supplementary Figure 15: As Figure 2 in the main text but here two versions of SURFBAT are compared where the test statistic is calculated either as a paired or unpaired test. Only the qq-plot of the unpaired test is given here in (b) as the qq-plot of the paired test can be found in Figure 2 in the main text. (c) Direct comparison of p-values between the two tests on the negative log scale.

**References**

Atkinson EG, Maihofer AX, Kanai M, Martin AR, Karczewski KJ, Santoro ML, Ulirsch JC, Kamatani Y, Okada Y, Finucane HK, et al. 2021. Tractor uses local ancestry to enable the inclusion of admixed individuals in GWAS and to boost power. Nat Genet. 53(2):195–204. doi:10.1038/s41588-020-00766-y.

Barc J, Tadros R, Glinge C, Chiang DY, Jouni M, Simonet F, Jurgens SJ, Baudic M, Nicastro M, Potet F, et al. 2022. Genome-wide association analyses identify new Brugada syndrome risk loci and highlight a new mechanism of sodium channel regulation in disease susceptibility. Nat Genet. 54(3):232–239. doi:10.1038/s41588-021-01007-6.

Baumdicker F, Bisschop G, Goldstein D, Gower G, Ragsdale AP, Tsambos G, Zhu S, Eldon B, Ellerman EC, Galloway JG, et al. 2022. Efficient ancestry and mutation simulation with msprime 1.0. Genetics. 220(3):iyab229. doi:10.1093/genetics/iyab229.
